# High-Entropy Engineering in Hollow Layered Hydroxide Arrays to Boost 5-Hydroxymethylfurfural Electrooxidation by Suppressing Oxygen Evolution

**DOI:** 10.1021/acscentsci.4c01085

**Published:** 2024-10-03

**Authors:** Yu Xin, Hongchuan Fu, Liyu Chen, Yongfei Ji, Yingwei Li, Kui Shen

**Affiliations:** † Guangdong Provincial Key Lab of Green Chemical Product Technology, School of Chemistry and Chemical Engineering, South China University of Technology, Guangzhou 510640, China; ‡ School of Chemistry and Chemical Engineering, Guangzhou University, Guangzhou 510006, China

## Abstract

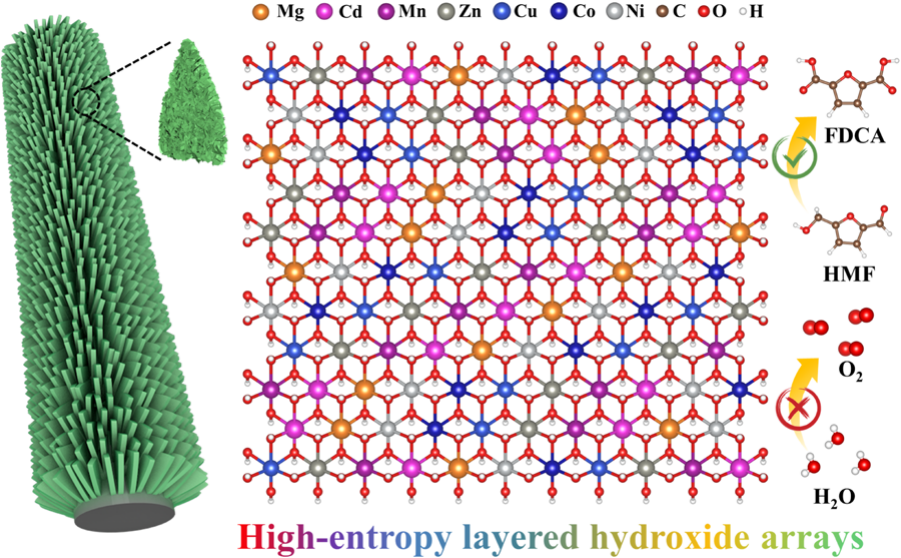

The electricity-driven 5-hydroxymethylfurfural (HMF) oxidation reaction has exhibited increasing potential to produce high-value-added 2,5-furandicarboxylic acid (FDCA). Unfortunately, the competitive oxygen evolution reaction (OER) can decrease the yield and Faradaic efficiency (FE) of FDCA under high potentials. Here, we report a general MOF-templated strategy to construct a new class of hollow high-entropy layered hydroxide array (HE-LHA) electrocatalysts including quinary, senary, and septenary phases composed of CoNiMnCuZn with Cd and Mg on carbon cloth (CC) for boosting the HMF oxidation reaction (HMFOR) by suppressing the OER. Impressively, the septenary CC@CoNiMnCuZnCdMg-LHA exhibits a low potential of 1.42 V_RHE_ for the HMFOR but a high potential of 1.68 V_RHE_ for the OER to achieve 100 mA cm^–2^, ranking it among one of the best electrocatalysts for the HMFOR. Finite element simulations show its hollow array morphology can induce a strong local electric field over all of the shell, thus favoring the electrocatalytic process. *In situ* electrochemical impedance spectroscopy and theoretical calculations further reveal that the Co, Ni, Cu, Zn, Mn, Cd, and Mg metals in high-entropy LHAs can accelerate the HMFOR but suppress the OER by optimizing the adsorption energy of the HMF* and OH*. This work sheds light on the rational design and construction of high-entropy nanoarchitectures for advanced electrocatalysis.

## Abstract 2

A general MOF-templated strategy is proposed to construct a new class of hollow high-entropy layered hydroxide array electrocatalysts for boosting HMF oxidation reaction by suppressing OER.

## Introduction

The rapid depletion of fossil fuels and increasingly serious environment problems have intensified the need for clean energy.^1,2^ Electrocatalytic overall water splitting (OWS) technology has been widely regarded as the most promising and sustainable way to produce clean and storable hydrogen energy with a high energy density of 120 MJ kg^–1^, which currently occupies ∼4% of the total hydrogen production in the global market.^3^ This process is composed of two half-reactions containing cathodic hydrogen evolution reaction (HER) and anodic oxygen evolution reaction (OER).^4,5^ However, the realization of a fully integrated water splitting system has been largely limited by the sluggish OER.^6,7^ Recently, replacing the OER with more thermodynamically favorable small molecule oxidation reactions (e.g., 5-hydroxymethylfurfural (HMF),^8−11^ urea,^12−15^ glucose,^16−18^ tetrahydroisoquinolines,^19−21^ hydrazine)^22−24^ is more in line with the concept of energy-saving development, among which the lignocellulose-derived HMF electrooxidation reaction (HMFOR) has been considered as one of the most fascinating reactions. This is because its oxidative product, 2,5-furandicarboxylic acid (FDCA), is an important platform molecule in the synthesis of biodegradable polyethylene 2,5-furandicarboxylate (PEF, polymerized from FDCA and ethylene glycol),^25−27^ which has been regarded as a promising candidate to replace the traditional polyethylene terephthalate (PET) polymer^28,29^ in the production of plastic products (Figure S1). Accordingly, coupling anodic HMFOR with cathodic HER can serve as an ecofriendly and economically viable pathway for FDCA synthesis, which endows enormous potential for both H_2_ and PEF production.

As electrochemical reactions always take place on the surface of electrocatalysts, the morphological structure of an electrocatalyst can strongly influence its activity. Particularly, the controlled synthesis of self-supported hollow array electrocatalysts has drawn great promise to boosting electrocatalysis due to their high surface-to-volume ratios and favorable mass diffusion properties. Among the various promising non-noble metal electrocatalyst candidates toward HMFOR, two-dimensional (2D) layered double hydroxides (LDHs) composed of two types of transition metals (e.g., Fe, Co, Ni, etc.) have been widely investigated.^30−33^ However, these electrocatalysts always have high intrinsic OER activity that would favor O_2_ evolution kinetics and thus cause a low Faradaic efficiency (FE) of FDCA under high potentials and large current densities.^34−36^ Additionally, the generated O_2_ bubbles over these electrocatalysts are detrimental to the adsorption of HMF molecules. For example, NiFe-LDH-based electrocatalysts have exhibited both superb HMFOR^30^ and OER performances^34^ due to the presence of bimetallic Fe–O–Ni active sites, which are actually not suitable for HMFOR under high potentials; thus, the applied potential range of NiFe-LDH-based electrocatalysts for HMFOR is greatly restricted.^37^ To suppress oxygen evolution and thus obtain a high FDCA FE, introducing more doped metal atoms with low OER activity (e.g., Cu, Mg, etc.) into LDHs may provide an effective way for boosting HMFOR.^8,27,38^

With the metallic element types in a single-phase LDH increasing to more than five, high-entropy layered hydroxides (HE-LHs) can be formed. As a new concept in the field of materials science, high-entropy materials (HEMs) have attracted tremendous interest in various fields, especially in catalysis.^39,40^ Benefiting from the abundant elemental composition in a single-phase structure, HEMs such as high-entropy alloys, oxides, hydroxides, and sulfides have exhibited great potential for efficient catalysis because the formed lattice distortion and metallic synergistic effect can better optimize the kinetic barrier for the adsorption and desorption of reaction intermediates as compared to traditional heterostructure catalysts.^41−43^ Theoretically, HE-LHs can form easily under high-temperature conditions according to the Gibbs free energy formula (Δ*G*_mix_ = Δ*H*_mix_ – *T*Δ*S*_mix_). However, the instability and phase transition of hydroxides at high temperatures have limited their synthesis.^41^ In addition, an extremely strong alkaline environment is also indispensable for the conventional codeposition method due to the huge difference in solubility product constants (*K*_sp_) of different metallic ions, in which the corresponding hydroxide products are usually disordered with thick layers and uncontrollable morphology.^44,45^ Unlike electrodeposition (needing high electricity consumption) and hydrothermal methods (needing high-temperature and strong alkaline conditions), the ion-exchange strategy used in this study can realize the controlled synthesis of HE-LHs especially with a favorable hollow array structure under both room temperature and a weak base environment.

In this work, a metal–organic framework (MOF)-templated strategy to construct a new class of hollow high-entropy layered hydroxide array (HE-LHA) electrocatalysts on carbon cloth (CC) is put forward. We have demonstrated that the septenary CC@CoNiMnCuZnCdMg-LHA electrocatalyst (denoted as CC@LHA(7)) can accelerate HMFOR but suppress the OER process since it only needs a low potential of 1.42 V vs RHE (V_RHE_) for HMF electrooxidation but a high potential of 1.68 V_RHE_ for OER to achieve a current density of 100 mA cm^–2^. Furthermore, CC@LHA(7) exhibits a high yield and high FE of FDCA (both nearly 100%), as well as good durability. To the best of our knowledge, this is the first successful application of high-entropy layered hydroxides for boosting HMFOR by suppressing oxygen evolution. Theoretical simulations show that the hollow leaf-like array morphology can induce a strong local electric field over all of the shell, which can favor the electrocatalytic process. In addition, *in situ* electrochemical impedance spectroscopy (EIS) and first-principles calculations also reveal that the Ni, Cu, Zn, Mn, Cd, and Mg metals in high-entropy LHAs can accelerate the HMFOR but suppress the OER. This study sheds light on the rational design and construction of high-entropy nanoarchitectures for advanced electrocatalysis.

## Results and discussion

### Preparation and Characterization of CC@LHAs(*n*)

**Figure 1:**
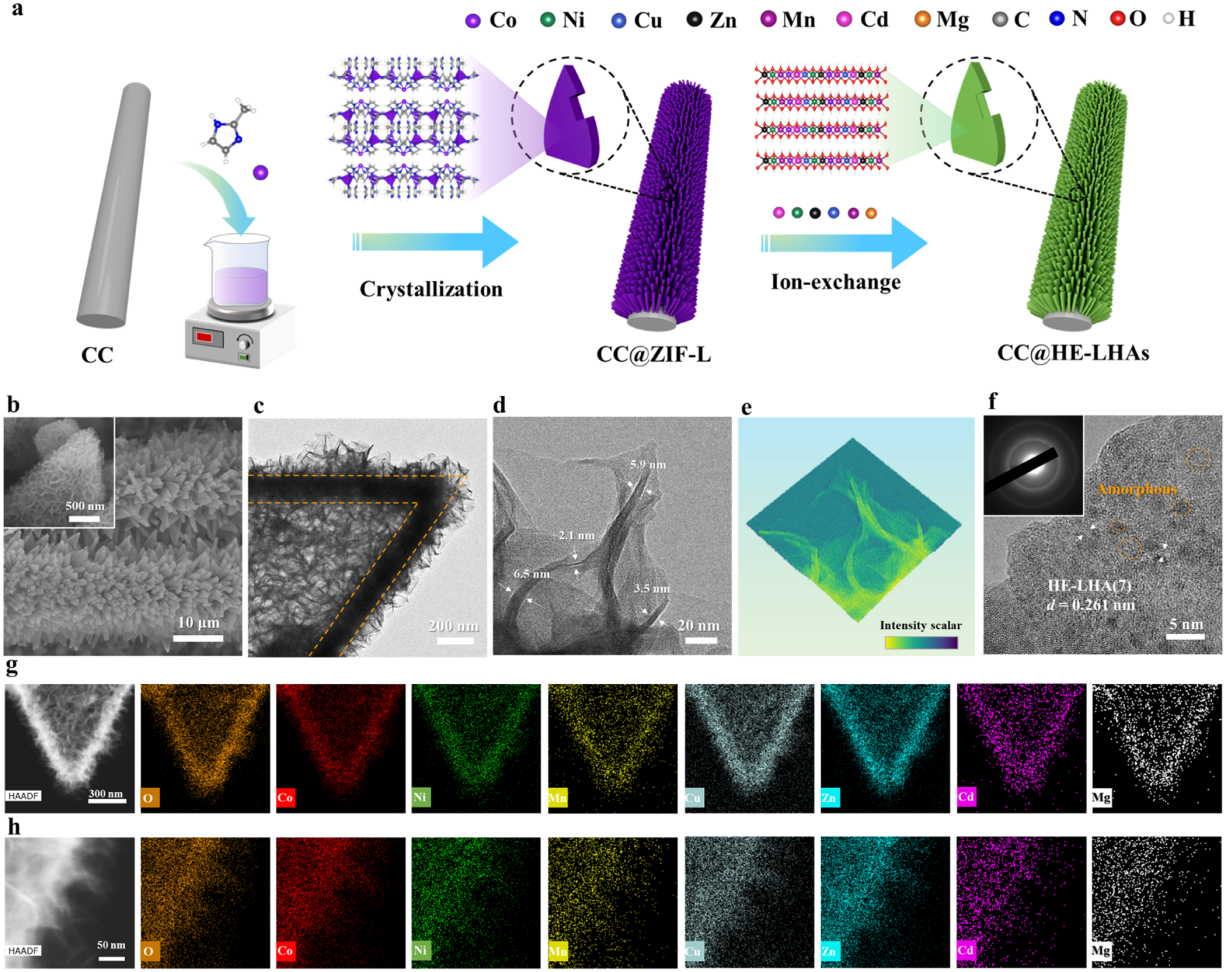
(a) Schematic illustration of the synthesis process of self-supported hollow HE-LHAs on CC (named CC@LHAs(*n*)). (b) SEM image, (c, d) TEM images, (e) corresponding 3D pseudocolor surface plot, and (f) HRTEM image of CC@LHA(7). (g, h) HAADF-STEM and corresponding elemental mapping images of CC@LHA(7), composed of seven dissimilar metallic elements, at two different magnifications. The insets in (b) and (f) are the high-magnification SEM image and the SAED image of CC@LHA(7), respectively.

Based on the Gibbs free energy formula, the influence of enthalpy may occupy the leading status under a low synthetic temperature, which may cause the enrichment of the same metal elements. Additionally, the different *K*_sp_ values of various metallic ions mean that a relative high pH value is required to form different hydroxides according to the *K*_sp_ formula (Figure S2 and Table S1). These may cause the local stress of the as-prepared HE-LHAs to rise to a high level, which may finally induce phase separation. Given the above problems, we designed a MOF-templated self-etching strategy to form a locally high pH interface on a solid surface by H^+^ consumption. As shown in Figure 1a, the HE-LHAs were successfully synthesized by a simple two-step method including crystallization and ion-exchange. First, uniform cobalt-based leaf-like ZIF-L arrays with smooth surfaces were directly grown on the flexible CC substrate (Figures S3 and S4) through a simple crystallization process by the chemical reaction between Co(NO_3_)_2_ and 2-methylimidazole (denoted as CC@ZIF-L). Subsequently, by immersing CC@ZIF-L into ethanolic solutions with different metal salts (Ni^2+^, Cu^2+^, Zn^2+^, Mn^2+^, Cd^2+^, and Mg^2+^), three-dimensional self-supported hollow arrays containing quinary, senary, or septenary HE-LHAs on CC surface were obtained (denoted as CC@LHAs(*n*), where *n* refers to the number of metal elements in HE-LHAs).

The morphology and structure of CC@LHA(7) were first investigated by scanning electron microscopy (SEM) and transmission electron microscopy (TEM). The SEM images of CC@LHA(7) reveal the formation of rough LHA sheets decorated on the leaf-shaped arrays as compared with the smooth surface of CC@ZIF-L and CC (Figure 1b). A sharp contrast between the bright core and the dark edge shell of a typical LHA(7) nanosheet can be clearly observed in its TEM image, which provides unambiguous evidence of the favorable hollow structure of the LHA(7) nanosheets (Figure 1c). The magnified TEM image and corresponding 3D pseudocolor surface plot of CC@LHA(7) clearly show its ultrathin nanolayers with thicknesses ranging from 2.1 to 6.5 nm (Figure 1d,e). The high-resolution TEM (HRTEM) image and its corresponding selected-area electron diffraction (SAED) further indicate that the selected LHA(7) nanosheet is polycrystalline, with some amorphous areas (yellow circles in Figure 1f). More importantly, the high-angle annular dark field scanning transmission electron microscopy (HAADF-STEM) and energy dispersive X-ray spectroscopy (EDS) elemental mapping images of CC@LHA(7) indicate that the seven metal elements and the O element are distributed randomly and uniformly throughout the hollow array structure with no phase separation (Figure 1g,h). All of these results unequivocally confirm the successful formation of the high-entropy layered hydroxide arrays of CC@LHA(7) with a hierarchical, self-supported, and hollow structure.

**Figure 2:**
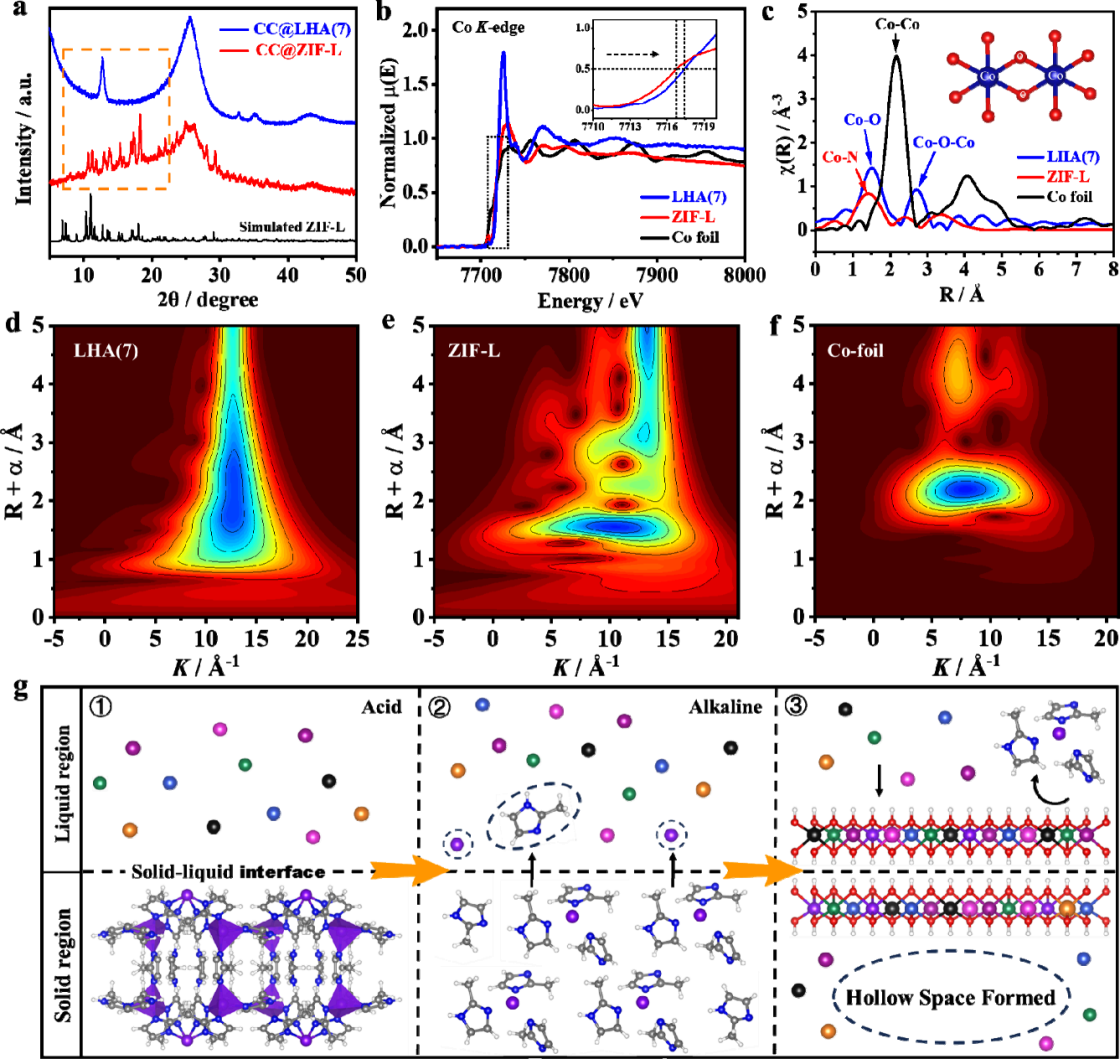
(a) XRD patterns of CC@ZIF-L and CC@LHA(7). (b) Normalized energies of absorption Co K-edge XANES (*E*_edge_) and (c) *k*^2^-weight Fourier transformation of Co K-edge EXAFS spectra of CC@ZIF-L, CC@LHA(7), and Co foil. The inset in (b) is the enlarged view corresponding to the black dotted line region. Wavelet transform (WT) contour maps for EXAFS *k*^2^χ(*k*) of (d) LHA(7), (e) ZIF-L, and (f) Co foil. (g) Kirkendall effect assisted ion-exchange mechanism in the synthesis of hollow HE-LHA(7).

As compared with that of CC@ZIF-L, the X-ray diffraction (XRD) pattern of CC@LHA(7) displays a new diffraction peak at 10–12°, which can be indexed to the (003) crystal plane of LHA(7), while the typical diffraction peaks of ZIF-L disappeared (Figure 2a). In addition, X-ray absorption fine structure (XAFS) spectra of CC@ZIF-L and CC@LHA(7) were obtained to investigate the transform mechanism from the ZIF structure to a layered hydroxide structure. In the X-ray absorption near edge structure (XANES) spectra of ZIF-L and LHA(7), remarkable features of electronic transitions from the core levels to unoccupied states are displayed. With the transformation from the ZIF-L precursor to LHA(7), the energy of the absorption Co K-edge (*E*_edge_) evaluated from the half height (0.5) of the normalized edge increases from 7716.7 to 7717.4 eV, indicating the enhanced specific oxidation state of LHA(7) as compared to ZIF-L (Figure 2b). Besides, the *k*^2^-weight Fourier transformation (FT) curves of Co K-edge extended X-ray absorption fine structure (EXAFS) can provide more detailed information about the coordination environment at the atomic level. It is found that both C–O and Co–O–Co bonds exist in the LHA(7) sample, but neither Co–Co nor Co–N can be observed (Figure 2c). The signals from wavelet transform plots for the Co EXAFS *k*^2^χ(*k*) of LHA(7), ZIF-L, and Co foil show the maximal intensities of 12.6, 11.2, and 7.6 Å^–1^, which can be contributed to Co–O, Co–N, and Co–Co scattering, respectively (Figure 2d–f). The Fourier transform infrared (FT-IR) spectrum of CC@LHA(7) also shows the disappearance of the C–N bond at 995 cm^–1^ and the N–H bond at 756 cm^–1^, indicating the complete removal of 2-methylimidazole ligands during the synthesis of CC@LHA(7) (Figure S5). All these results coherently confirm the successful transformation from the Co–N coordinated ZIF-L structure to the Co–O coordinated hydroxide structure. On the basis of our results as well as the previous advances in this field,^7,40,45^ we can conclude that the formation of hollow arrays in our study involves a dissolution–regrowth process based on the classical Kirkendall effect, in which the regrowth rate is lower than the dissolution rate (Figure 2g). To be more specific, the acid-labile ZIF-L precursor can be easily decomposed in the weakly acidic ethanolic solution formed by the hydrolysis of metal salts (step ① in Figure 2g). With the consumption of H^+^ ions, the framework of ZIF-L is decomposed, and thus, free Co^2+^ ions and 2-methylimidazole molecules are released on the sold–liquid interface, which can lead to the increase of the local pH value (step ② in Figure 2g). Finally, the metal ions (Ni^2+^, Cu^2+^, Zn^2+^, Mn^2+^, Cd^2+^, and Mg^2+^ ions) in solution as well as the Co^2+^ ions on the ZIF-L surface can coprecipitate with the OH^–^ ions to form a hollow array structure with HE-LHA nanosheets decorated on the array surface (step ③ in Figure 2g). This strategy has broken through the limitation that various metal ions with large atomic radius differences have difficulty in forming HE-LHA, and even Cd^2+^ ions in the fourth period can be incorporated into the lattice of the hollow LHAs.

**Figure 3:**
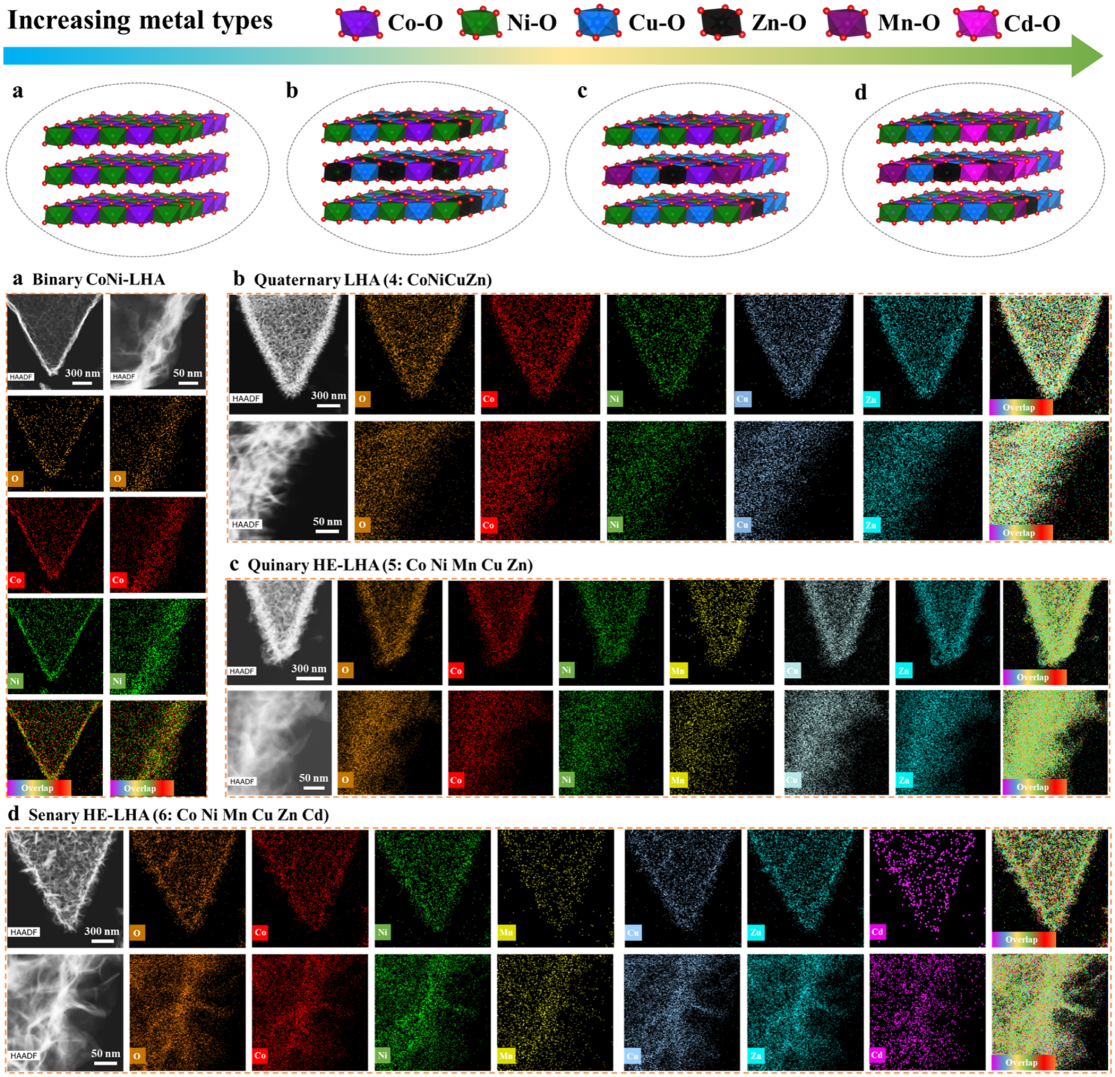
HAADF and STEM elemental mapping images of (a) binary (Co and Ni), (b) quaternary (Co, Ni, Cu, and Zn), (c) quinary (Co, Ni, Mn, Cu, and Zn), and (d) senary (Co, Ni, Mn, Cu, Zn, and Cd) CC@LHAs(*n*) arrays.

Encouragingly, we have successfully extended this versatile MOF-templated strategy to synthesize other types of CC@LHAs(*n*) with binary, quaternary, quinary, and senary metallic elements by controlling the number of metal elements (*n*) in the ion-etching step (see details in the Supporting Information), demonstrating the good generality of this developed methodology involving Kirkendall effect assisted ion-exchange. As shown in Figures S6–S10, all the as-prepared samples have a leaf-like array morphology and a hierarchical hollow structure, which are similar to those of CC@LHAs(7). In addition, as the number of metal element types increases from two (Co and Ni) to four (Co, Ni, Cu, and Zn), five (Co, Ni, Cu, Zn, and Mn), six (Co, Ni, Cu, Zn, Mn, and Cd), and seven (Co, Ni, Cu, Zn, Mn, Cd, and Mg), uniform distributions of all elements in the hollow structures of the resultant CC@LHAs(*n*) samples can be confirmed by their HAADF-STEM images and corresponding EDS elemental mapping images (Figure 1g,h and Figure 3), on which neither phase separation nor elemental segregation phenomena can be observed.

As for the crystal structures of various CC@LHAs(*n*), different shifts of the (003) plane peaks can also be clearly observed when various metal cations are doped into the LHAs, indicating that some differences exist in the interlayer spacings of these samples (Figure S11). The interlayer spacing of CoNiCuZn-LHA(4) determined by Bragg’s law is 8.1 Å, which has decreased a little as compared with that of CoNi-LHA(2) (8.5 Å). When the mixing entropy of CC@LHAs(*n*) increases to a certain extent (more than five types of metal elements exist), the interlayer spacing for HE-LHA(5), HE-LHA(6), and HE-LHA(7) decreases to 6.9, 6.8, and 6.8 Å, respectively. Note that only a small interlayer spacing difference can be observed between CoNi-LHA(2) and CoNiCuZn-LHA(4) because there is no larger metal cation than Co^2+^. However, the interlayer spacings of HE-LHA (5), HE-LHA(6), and HE-LHA(7) just increase a little, which may be caused by the doped Mn^2+^ and Cd^2+^ cations that have a large ionic radius. According to the XRD standard spectra of various hydroxides, Co-, Ni-, Zn-, Mn-, Cd-, and Mg-based hydroxides all have a *P*3̅*m*1 space group, but Cu(OH)_2_ has a *Cmcm* space group (Table S2). Although the lattice constants of Cu-based hydroxide are slightly different from those of the other hydroxides, the ionic radius of Cu^2+^ (0.73 Å) under a high-spin 6 coordination condition (very common for hydroxide structures) is similar to the arithmetic mean (*x̅* = 0.77 Å) of the above metal cations (Ni^2+^: 0.69 Å, Mg^2+^: 0.72 Å, Cu^2+^: 0.73 Å, Zn^2+^: 0.74 Å, Co^2+^: 0.75 Å, Mn^2+^: 0.83 Å, and Cd^2+^: 0.95 Å) (Figure S12 and Table S3).^41^ Therefore, the as-formed CC@LHAs(*n*) may have a relatively low structural stress, which could avoid the local ordered enrichment of the same elements in LHAs(*n*).

**Figure 4:**
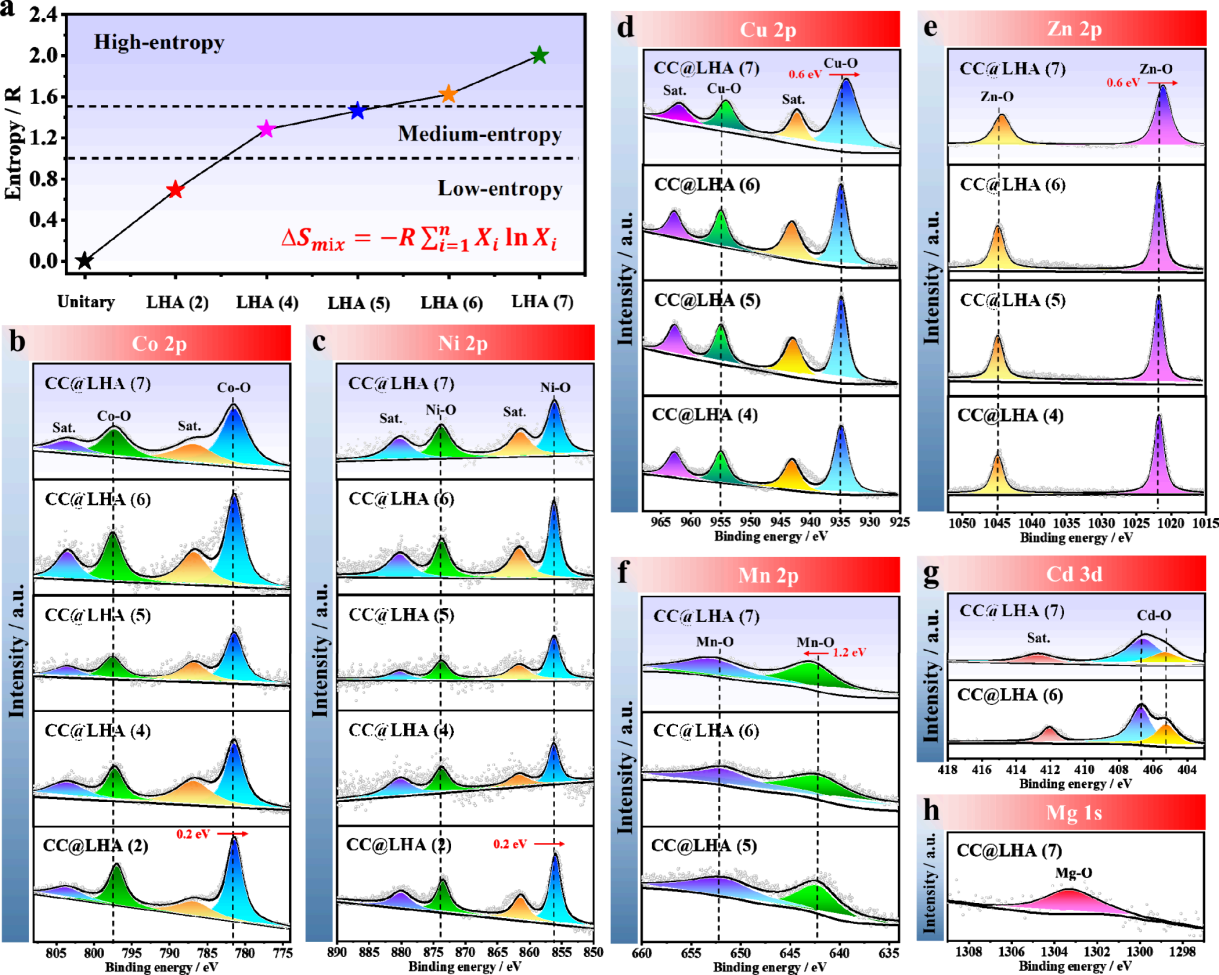
(a) Calculated configurational entropies of various CC@LHAs(*n*). High-resolution XPS spectra of (b) Co 2p, (c) Ni 2p, (d) Cu 2p, (e) Zn 2p, (f) Mn 2p, (g) Cd 3d, and (h) Mg 1s of various CC@LHAs(*n*) with different configurational entropies.

High-resolution X-ray photoelectron spectroscopy (XPS) measurements were conducted on various CC@LHAs(*n*) samples, in which almost all of the metal cations exhibit a divalent valence state (Figure 4). As expected, the XPS spectrum of CC@LHA(7) reveals the presence of seven metal elements containing cobalt (Co 2p), nickel (Ni 2p), copper (Cu 2p), zinc (Zn 2p), manganese (Mn 2p), cadmium (Cd 3d), and magnesium (Mg 1s). In the high-resolution Co 2p spectrum of CC@LHAs(7), the characteristic peaks at 797.2 and 781.5 eV can be assigned to the Co 2p_1/2_ and Co 2p_3/2_ tracks of the Co–O bonds, respectively (Figure 4b). Two satellite peaks at 803.5 and 786.7 eV can be observed in the Co XPS spectrum. The Ni 2p XPS spectrum presents the 2p_1/2_ and 2p_3/2_ characteristic peaks at 873.7 and 856.2 eV, respectively, corresponding to the Ni–O bonds in the LHA unit (Figure 4c). Two shakeup satellites at 880.2 and 861.7 eV can also be observed in the Ni XPS spectrum. The Cu 2p XPS spectrum shows that the characteristic peaks at 954.7 and 934.1 eV correspond to the Cu 2p_1/2_ and Cu 2p_3/2_ tracks of the Cu–O bonds (Figure 4d). Similar to the XPS spectra of Co and Ni, that of Cu also exhibits two satellite peaks at 962.5 and 942.8 eV. Additionally, the Zn 2p_1/2_ and Zn 2p_3/2_ orbital modes are situated at 1045.2 and 1022.2 eV without a shakeup satellite, which are attributed to the presence of Zn–O bonds (Figure 4e). The Mn 2p XPS spectrum shows 2p_1/2_ and 2p_3/2_ peaks at 652.1 and 642.6 eV, respectively, which are attributed to the Mn–O bonds (Figure 4f). The Cd 3d XPS spectrum shows spin–orbit pairs at 406.2 eV (Cd 3d_5/2_) and 412 eV (Cd 3d_3/2_), which can be separated into Cd–O bonds (Figure 4g). The binding energy of 1303.6 eV can be attributed to Mg–O bonds in the Mg 1s XPS spectrum (Figure 4h). It is noteworthy that the binding energies of both Co 2p and Ni 2p in CC@LHA(2) shift by about 0.2 eV to lower energies compared with those of other CC@LHAs (Figure 4b,c), indicating that the partial electrons of Ni^2+^ with a fully filled t_2g_ orbital and Co^2+^ with a t_2g_ orbital in a high-spin state can easily transfer to other metal ions by the strong O^2–^ bridge mediated π-donation interaction in CC@LHA(4), CC@LHA(5), CC@LHA(6), and CC@LHA(7), while the further addition of Mn, Cd, and Mg in CC@LHA(5), CC@LHA(6), and CC@LHA(7) has little effect on the electrons of Co or Ni (Figure S13). Additionally, the binding energies of Mn 2p in CC@LHA(7) shift by about 1.2 eV to higher energies, while the binding energies of both Zn 2p and Cu 2p shift by about 0.6 eV to lower energies compared with those of other CC@LHAs (Figure 4d–f), which reveals that the addition of Mg can also induce the relaxation effect caused by O^2–^ bridge mediated interatomic electron transfer.^43^ Additionally, the addition of Mn, Cd, and Mg in CC@LA(5), CC@LHA(6), and CC@LHA(7) has little effect on the electrons of Co and Ni (Figure 4b,c), while the addition of Mg in CC@LHA(7) can affect the electron distributions of Cu, Zn, and Mn (Figure 4d–f), in which some metal atoms can act as electron donors (positive shift) or electron acceptors (negative shift) to other metal atoms. To understand the charge transfer in LHA(7), we also calculate the Bader charges of the metal ions in LHA(2), LHA(6), and LHA(7) based on density functional theory (DFT) calculations. The models and results are shown in Figure S14 and Table S4. We can see that the difference in the charge is rather small. Comparing LHA(7) to LHA(6), the Mn ion becomes more positively charged by 0.117 e, while Cu and Zn become less positively charged by 0.01 e and 0.03 e, which agrees well with the scale and direction of the shift in binding energies. For Co and Ni, the XPS spectra reveal a shift of 0.2 eV in the binding energy from LHA(2) to LHA(7), which is much smaller than that for Cu (0.6 eV), Zn (0.6 eV), and Mn (1.2 eV). Therefore, the change in the Bader charge is expected to be much smaller. As shown in Table S4, the differences in charge of Co and Ni in LHA(2), LHA(6), and LHA(7) are within 0.01 e, which agrees qualitatively with the XPS results.

As high-entropy engineering has been successfully introduced into the two-dimensional LHAs, the formula of the resultant HE-LHAs(*n*) can be concluded as , where *n* is the number of the metal elements, M^*x*+^ is the divalent or trivalent metal cations, and A^*y*–^ is the interlayer anions. The as-prepared CC@LHAs(*n*) also follow Pauling’s rule^44^ because the amount of divalent metal cations (M^2+^) is at least twice that of the trivalent metal cations (M^3+^) in LHAs (the molar ratio of M^2+^ and M^3+^ is >2) based on the XPS results. According to the Boltzmann entropy equation^43,46^ of  (where *R* is the ideal gas constant and *x*_*i*_ represents the mole fraction of the *i*th component), the configuration entropy of a high-entropy material is always larger than 1.5*R* in a random state, while materials with Δ*S* between 1.0*R* and 1.5*R* and with Δ*S* < 1.0*R* are usually classified as medium-entropy and low-entropy materials, respectively.^43^ Based on the above equation and classification, the configuration entropy of CC@LHA(2), CC@LHA(4), CC@LHA(5), CC@LHA(6), and CC@LHA(7) is calculated to be 0.69*R*, 1.28*R*, 1.46*R*, 1.62*R*, and 2*R*, respectively (Figure 4a). Obviously, the CC@LHA(6) and CC@LHA(7) samples can be classified into high-entropy materials, while the CC@LHA(2), CC@LHA(4), and CC@LHA(5) samples are classified into low- or medium-entropy materials in our study.

### HMFOR Performance

**Figure 5:**
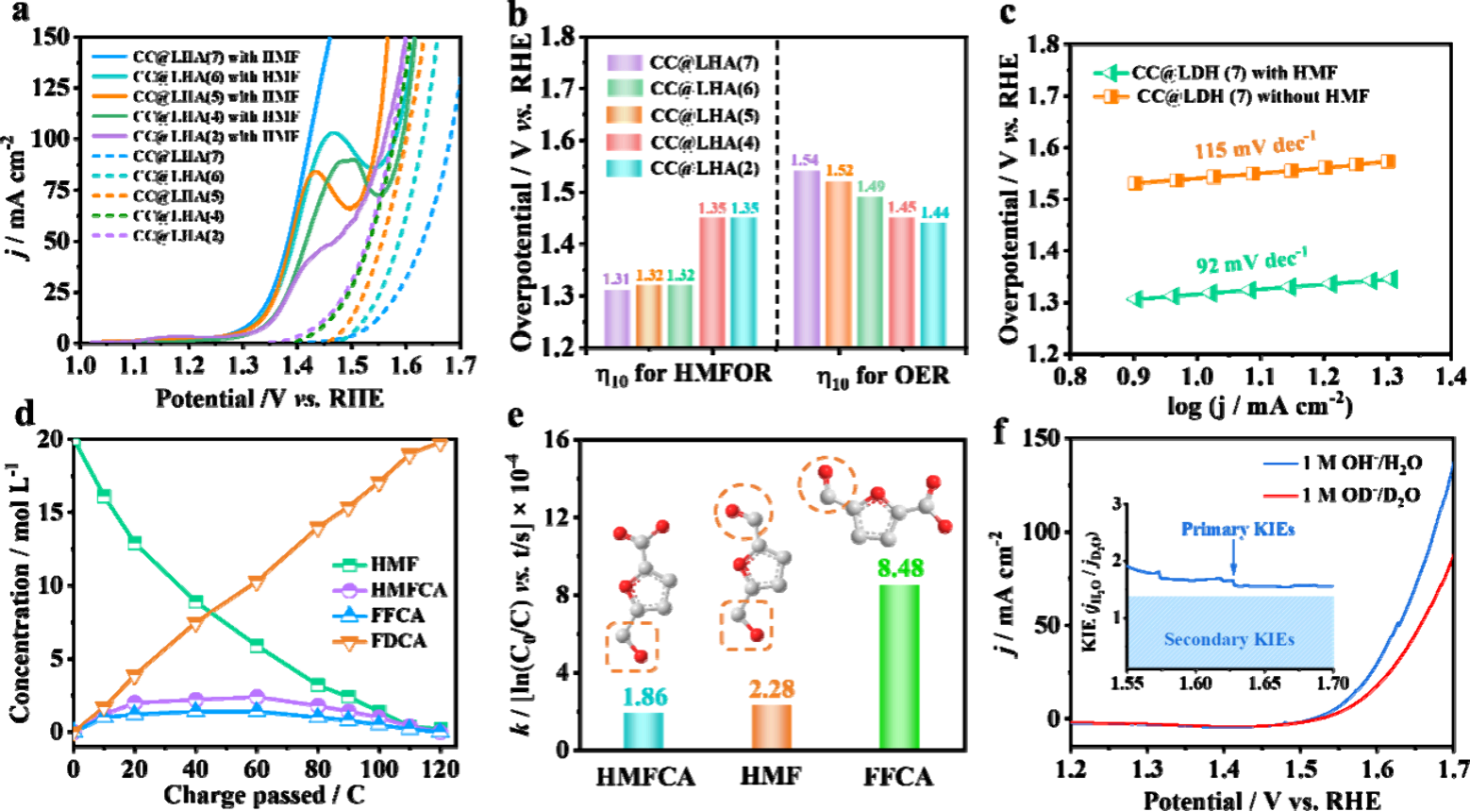
(a) LSV curves and (b) potential comparison of HMFOR and OER over various CC@LHAs(*n*) samples to reach the same current density of 10 mA cm^–2^. (c) Tafel slopes of CC@LHA(7) with and without HMF. (d) Concentration evolutions of HMF, FDCA, and various intermediates in 1 M KOH containing 20 mM HMF at 1.45 V vs RHE determined by HPLC. (e) The pseudo-first-order kinetic constants of HMFCA, HMF, and FFCA electrooxidation. (f) LSV curves of CC@LHA(7) in 1 M OD^–^/D_2_O or 1 M OH^–^/H_2_O.

Subsequently, the electrocatalytic performances of various samples for HMFOR and OER were explored in a standard three-electrode alkaline system (1 M KOH) in the presence or absence of 20 mM HMF. Given that the OER is the main competitive reaction during the HMFOR process, we provide the linear sweep voltammetry (LSV) curves of CC@LHAs(*n*) for the HMFOR and OER in Figure 5a. Typically, CC@LHA(7) shows a small onset potential at ∼1.35 V_RHE_ for the HMFOR (solid black line) and ∼1.45 V_RHE_ for the OER (dotted black line). The current density of HMFOR for CC@LHA(7) can reach 100 mA cm^–2^ at 1.42 V_RHE_, which is even lower than the onset potential of OER. The HMFOR potentials of CC@LHAs(*n*) exhibit a decreasing tendency from 1.35 V_RHE_ for CC@LHA(2) to 1.31 V_RHE_ for CC@LHA(7) at a current density of 10 mA cm^–2^, all of which are much lower than their corresponding potentials for the OER to reach the same current density (Figure 5b and Figure S15). In sharp contrast, the OER potentials of the above electrocatalysts exhibit an increasing tendency, which are 1.44, 1.45, 1.49, 1.52, and 1.54 V_RHE_ for CC@LHA(2), CC@LHA(4), CC@LHA(5), CC@LHA(6), and CC@LHA(7) to reach the same current density of 10 mA cm^–2^, respectively. After the incorporation of Mn, Cd, and Mg, the decreased OER and enhanced HMFOR electrocatalytic performances of CC@LHA(5), CC@LHA(6), and CC@LHA(7) can be clearly observed as compared with those of CC@LHA(2) and CC@LHA(4), indicating that the synergistic effects of Mg, Cd, and Mg elements can adjust the electronic structures of hydroxides and thus enhance the catalytic activity for the HMFOR. In addition, the Tafel slope of CC@LHA(7) for HMFOR (92 mV dec^–1^) is lower than that of OER (115 mV dec^–1^), indicating the remarkably enhanced HMFOR but sluggish OER kinetics over this electrocatalyst (Figure 5c).

HMFOR usually contains the cleavage of C–H and O–H bonds as well as their corresponding deprotonation and electron transfer processes. To demonstrate the high selectivity of FDCA product during the HMFOR process, the HMFOR experiment was performed in an H-type cell set at 1.45 V_RHE_ catalyzed by the CC@LHA(7). The concentrations of reactant, intermediates, and products were detected after every 25 C by high-performance liquid chromatography (HPLC). As shown in Figure 5d and Figures S16 and S17, the yield and FE of FDCA can reach 99.99% and 99.05%, respectively, and almost no O_2_ is generated with a near 100% carbon balance. Figure S18 shows the FE of FDCA for CC@LHA(2) and CC@LHA(7) under various potentials in a 20 mM HMF solution. Impressively, CC@LHA(7) can maintain a high FE plateau across a wide potential range, which further indicates its good inertness to the OER. In contrast, the FE of FDCA for CC@LHA(2) drops dramatically in the potential range above 1.60 V_RHE_ since its significant OER activity could remarkably hamper its HMFOR performance. During this electrooxidation process, only 5-hydroxymethylfurancarboxylic acid (HMFCA) and 5-formyl-2-furancarboxylic acid (FFCA) appeared as intermediates instead of 2,5-diformylfuran (DFF) (Figure S19). This is because the aldehyde group (−CHO) on HMF is easier to be oxidized to a carboxylate group (−COOH) than the hydroxymethyl group (−CH_2_OH) in an alkaline electrolyte. From a kinetics view, all of the electrooxidation reactions of HMF, HMFCA, and FFCA follow a pseudo-first-order reaction (Figure S20). The oxidation rate constants of FFCA (8.48 × 10^–4^) and HMF (2.28 × 10^–4^) are obviously larger than that of HMFCA (1.86 × 10^–4^), indicating that the oxidation of −CH_2_OH to −CHO is the rate-determining step (RDS) during HMFOR (Figure 5e).

To investigate the RDS and proton transfer passivation process of the OER, deuterium kinetic isotope effects (KIEs) experiments were performed in a deuterated aqueous solution (1 M NaOD in D_2_O) or a protonated aqueous solution (1 M NaOH in H_2_O). The utilization of a deuterated solution as an electrolyte can effectively slow the proton transfer kinetics as compared with using a protonated aqueous solution as an electrolyte. The primary KIE value of CC@LHA(7) is calculated to be ∼1.6, further confirming that the cleavage of the O–H bond is the RDS during the OER (Figure 5f). Electrochemical impedance spectroscopy (EIS) tests were also conducted in 1 M OH^–^/H_2_O or OD^–^/D_2_O. According to the Nyquist plots of CC@LHA(7) at 1.55 V_RHE_, both the series resistance (*R*_s_) and the charge transfer resistance (*R*_ct_) in protonated OH^–^/H_2_O electrolyte (*R*_s_ = 1.14 Ω, *R*_ct_ = 0.87 Ω) are lower than those in deuterated OD^–^/D_2_O electrolyte (*R*_s_ = 1.29 Ω, *R*_ct_ = 1.10 Ω) (Figure S21), which reveals that the cleavage of the O–D bond is harder than that of the O–H bond, as also evidenced by the KIEs results.

**Figure 6:**
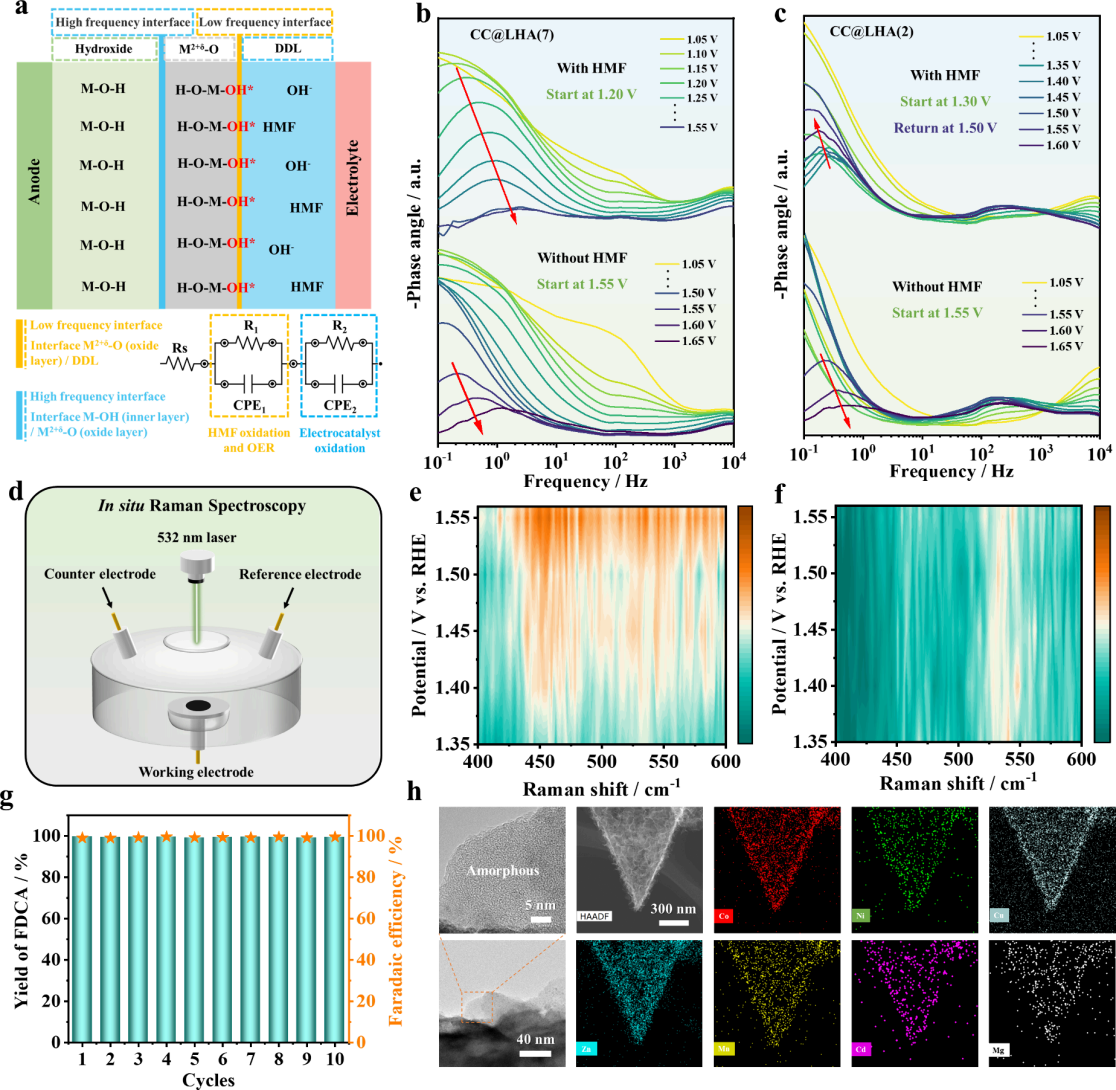
(a) Schematic diagram for the relationships between surface hydroxide/hydroxyl oxide species, interfaces, and reactants (H_2_O and HMF). Bode phase plots of the *in situ* EIS in the presence or absence of HMF on (b) CC@LHA(7) and (c) CC@LHA(2). (d) Schematic diagram of *in situ* Raman spectroscopy. *In situ* 2D Raman spectra of CC@LHA(7) in (e) 1 M KOH and (f) 1 M KOH with 20 mM HMF. (g) Stability test over CC@LHA(7) within 10 cycles for HMFOR. (h) HRTEM, HAADF-STEM, and elemental mapping images of CC@LHA(7) after the electrocatalytic test.

In addition to kinetic tests, *in situ* techniques have also been widely used to explore the intrinsic mechanism during the electrocatalytic process. We performed *in situ* EIS tests to clarify the interfacial evolution of various electrocatalysts between the electrode and electrolyte. The equivalent circuit model was employed to match the different potentials during the HMFOR or OER according to the Nyquist and Bode plots (Figure 6a–c and Figure S22). For CC@LHA(7) in 1 M KOH without adding HMF, the signals in the Bode plots located at the high-frequency interface (10^1^–10^3^ Hz) indicate the electrooxidation formation of surface hydroxide/hydroxyl oxide species, and the characteristic peaks of the OER starting at 1.55 V_RHE_ occur at the low-frequency interface (10^–1^–10^1^ Hz) (Figure 6b). After the addition of HMF, the peaks in the low-frequency region can be observed at lower potentials (1.20 V_RHE_), and the peaks for the OER are not evident. In contrast, as shown in Figure 6c, though CC@LHA(2) also manifests a similar trend of characteristic peaks in Bode plots as compared with CC@LHA(7) in KOH aqueous solution, the phase angles at the low-frequency interface exhibit a tendency of decreasing first and then increasing in the presence of HMF. Based on the potential consistency between LSV curves and *operando* EIS tests (Figures 5a and 6b,c), the strong reaction competitiveness of the OER can be clearly observed on CC@LHA(2) with the presence of HMF. Impressively, this HMF passivation phenomenon cannot be observed on CC@LHA(7), which also demonstrates the high FE of FDCA (99.99%) by suppressing the OER process on CC@LHA(7).

*In situ* Raman spectroscopy was also carried out to explore the surface structure evolution of CC@LHA(7) during the HMFOR and OER processes (Figure 6d). As shown in Figure 6e, a pair of Raman peaks at about 450 and 550 cm^–1^ can be assigned to the E_g_ bending vibration mode and the A_1g_ stretching vibration modes of Ni(III)–O in Ni–OOH species in CC@LHA(7), respectively. This pair of metal (oxy)hydroxide peaks is intensified significantly when the applied potential is increased to 1.45 V_RHE_, which can be attributed to the occurrence of the OER (Figure 6e and Figure S23a). In contrast, these characteristic peaks for metal (oxy)hydroxides have no significant enhancement in the presence of HMF, demonstrating that the generated M^2+δ^–O species can act with HMF molecules spontaneously with almost no occurrence of the OER (Figure 6f and Figure S23b). In addition, periodic electrochemical tests were also recorded to confirm this viewpoint. In the multipotential step curves of CC@LHA(7), the potential was switched from 1.4 to 1.0 V_RHE_ with injecting (blue line) and not injecting (red line) HMF in 1 M KOH (Figure S24). An extremely small anodic current (<1 mA cm^–2^) is observed under 1.45 V_RHE_ in 1 M KOH without HMF, while an obvious reduction current (∼30 mA cm^–2^) emerges when the applied potential is changed to 1.10 V_RHE_. This cathodic current can be ascribed to the electroreduction of the M^2+δ^–O species. In contrast, this reduction current can be decreased by injecting HMF into the electrolyte before switching the potential to 1.10 V_RHE_, indicating that the formed M^2+δ^–O can react with HMF spontaneously. In such a case, the CC@LHA(7) electrocatalyst can actually serve as a redox mediator via the reversible transformation between M^2+^–OH and M^2+δ^–O according to the indirect electrooxidation mechanism shown in Figure S25, while the activated Ni(OH)O intermediate is the activity origin for HMFOR and the doping of other metals also makes a contribution to multi-site synergistic catalysis by tuning the ligand environment of lattice oxygen (Ni–OH). Thus, the HMFOR process containing a two-step reaction with an electrogenerated catalytic dehydrogenation process and a spontaneous *HMF dehydrogenation process on CC@LHA(7) can be favorably accelerated.

Moreover, the electrocatalytic HMFOR performance over CC@LHA(7) remains almost unchanged over 10 successive cycles with good durability (Figure 6g). In addition, the long-term stability of CC@LHA(7) was evaluated by using a chronoamperometry consecutive recycling test (Figure S26), which was performed at 1.45 V_RHE_ in 50 mM HMF solution for 10 h with CC@LHA(7) as an anode. After the electrolyte was refreshed, HMF electrooxidation for another 10 h was conducted. During each 10 h electrolysis process, the current density decreased with prolonged reaction time, but it increased to the original level again after the electrolyte was refreshed. The chronoamperometry curve of each cycle kept almost the same decreasing current density tendency, which could be attributed to the gradually reduced HMF concentration (Figure S26). Overall, the steady operation of HMFOR over CC@LHA(7) has demonstrated its long-term stability. In addition, the gram-scale FDCA product (0.8 g) can be collected by acidifying the electrolytes after the continuous chronoamperometry test (Figures S27 and S28). The hollow nanoarchitecture of CC@LHA(7) remains unchanged after the electrocatalytic test, as proved by the SEM and TEM images in Figure S29. Additionally, the Co, Ni, Cu, Zn, Mn, Cd, and Mg elements are still uniformly distributed in the element mapping images and XPS spectra of the used CC@LHA(7) electrocatalyst, indicating the maintenance of its high-entropy characteristic (Figure 6h and Figure S30). The contents of different elements in CC@LHA(7) before and after the electrocatalytic test have no obvious changes except for the Zn element. Only a small amount of Zn (0.74 ppm) can be detected in the electrolyte after the test, which may be caused by its surface reconstruction during the electrocatalytic process (Table S5). It is noteworthy that the generation of an amorphous metal hydroxide layer can also be confirmed by the HRTEM images of the used CC@LHA(7), indicating this high-entropy LHA electrocatalyst may undergo a surface reconstruction process in the electrooxidation process (Figure S31). The (003) crystal plane diffraction peak disappeared in the XRD pattern of the used CC@LHA(7), which also validates the formation of an amorphous structure in this sample after the electrooxidation process (Figure S32). The O 1s XPS spectra of the fresh and used CC@LHA(7) clearly reveal that the content of oxhydryl-O (531.6 eV) is significantly increased as compared with that of lattice-O (530.5 eV) after the electrocatalytic test (Figure S33). In addition, the HMFOR performance of CC@LHA(7) was compared with those of various previously reported electrocatalysts, as summarized in Table S6, which confirms its outstanding catalytic activity and FDCA selectivity. Inspired by the excellent HMFOR performance of CC@LHA(7), we further evaluated its potential application in an HMFOR coupled HER two-electrode electrolyzer (Figure S34). The LSV curves of such an HMFOR coupled HER electrolyzer exhibit a lower cell voltage of 1.34 V as compared with that of the corresponding overall water splitting cell (1.62 V) at a current density of 10 mA cm^–2^ (Figure S35a). Moreover, the calculated electricity consumption of the HMFOR coupled two-electrode electrolyzer is measured to be 3.5 kW h to produce a cubic meter of H_2_ at a current density of 50 mA cm^–2^, which is also lower than 4.1 kW h for the corresponding overall water splitting cell (Figure S35b).

### Theoretical Calculation

**Figure 7:**
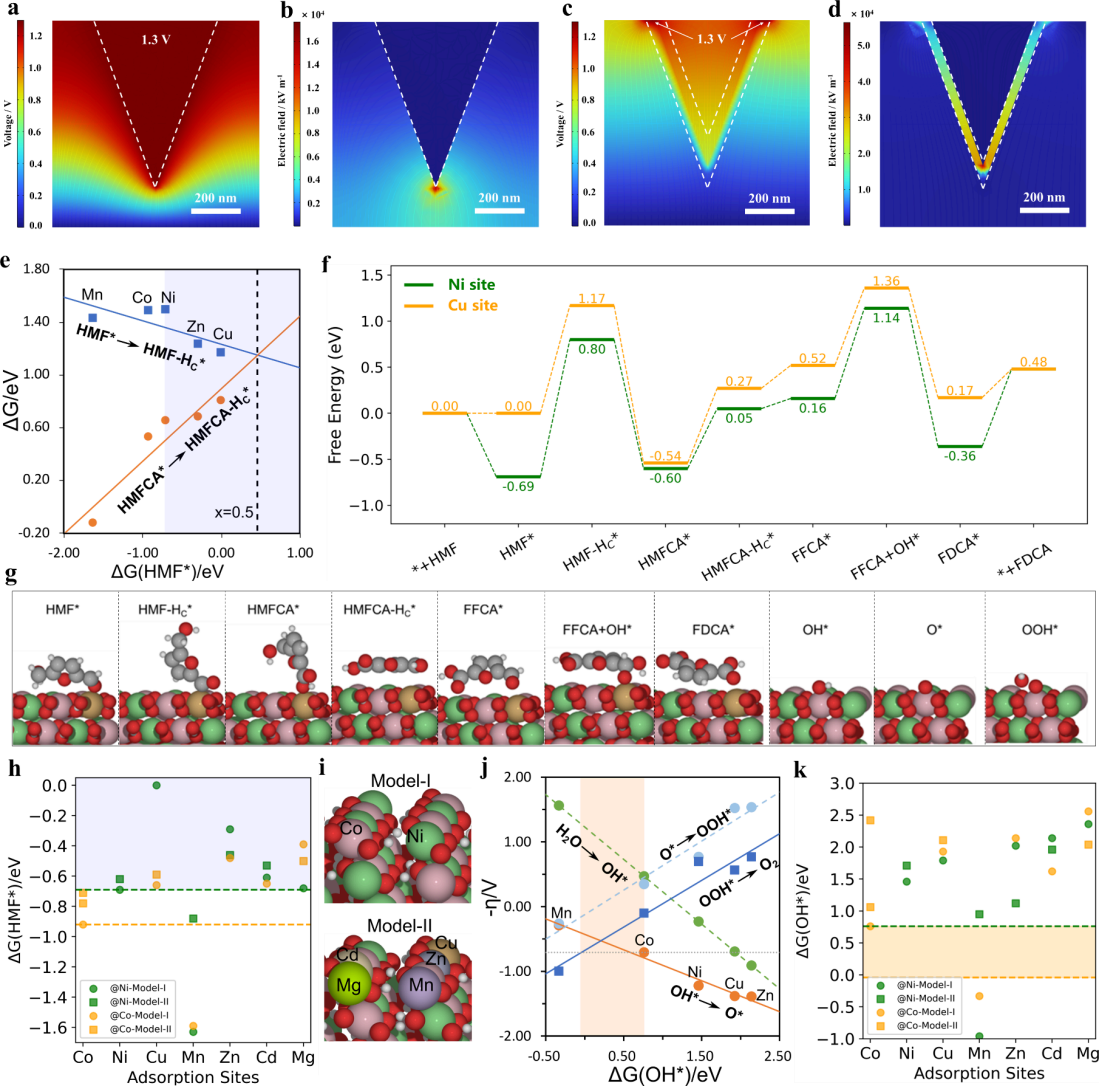
Simulated electric potential distribution and corresponding electric field distribution images of the (a, b) solid and (c, d) hollow leaf-like arrays. (e) The activity volcano for HMFOR based on model I. (f) The free energy diagrams for HMFOR at the Ni site of (Co, Ni)–OOH and Cu site of (Cu, Co, Ni)–OOH. (g) Typical adsorption structures of the intermediates on (Cu, Co, Ni)–OOH and (Co, Ni)–OOH. (h) The Δ*G*(HMF*) of the active sites in models I and II. (i) The molecule structure of models I and II. (j) The activity volcano for OER based on model I. (k) Δ*G*(OH*) of the active sites in models I and II (@Co/Ni means the Co/Ni site or the metal doped at the Co/Ni site).

Finite element simulations were first performed to reveal the advantages of the hollow leaf-like array morphology of the CC@LHA(7) electrocatalyst. First, a hollow leaf-shaped model and corresponding solid counterpart were established based on the STEM images, while a potential of 1.3 V was applied on the bottom of the arrays (Figure 7a,c). The electric field distribution images exhibit that the protons can accumulate around the nanotip of the solid array with a maximal electric field intensity of 1.77 × 10^4^ kV m^–1^ (Figure 7b), while protons can accumulate almost all over the hollow shell with an electric field intensity >2.0 × 10^4^ kV m^–1^ (Figure 7d). These results indicate that the hollow leaf-like array structure can break the limit of “nanotip-induced electric field”^47^ and promote the proton transfer activity over all of the hollow shell.

Subsequently, first-principles calculations based on DFT were performed to explore the HMFOR activity of (Co, Ni)–OOH. The calculated results reveal that the Ni and Co sites in the (Co, Ni)–OOH model have comparable activity (Figure 7e). Then, we doped the Ni site with Mn, Cu, and Zn to establish the scaling relations between the energies of the intermediates with the free energy of HMF* Δ*G*(HMF*) as the descriptor.^48^ The scaling relations are summarized in Figures S36–S39. First, we found that the HMF is preferentially adsorbed via the aldehyde group (Figure S36), which explains why the reactions proceed via the HMFCA path (HMF → HMFCA → FFCA → FDCA). Second, we found that in a wide range of Δ*G*(HMF*), HMF–H_C_* is preferred over HMF–OH*. For HMF → HMFCA, HMFCA–H_C_* is more favorable than HMFCA–H_O_* for HMFCA → FFCA, while FFCA+OH* is more stable than FFCA–H_C_* for FFCA → FDCA (Figure S37). Therefore, the reaction follows the path HMF* → HMF–H_C_* → HMFCA → HMFCA–H_C_* → FFCA → FFCA+OH* → FDCA*. Third, an activity volcano was constructed based on the scaling relation (Figure 7e), which shows that the reaction will be limited either by the oxidation of HMF or by the oxidation of HMFCA. The Mn doped site binds HMF* too strongly, which results in lower activity, while other atoms doped at the Ni site weaken the binding of HMF*, which results in higher activity. Among the Mn, Cu, and Zn doped cases, the Cu site exhibited the highest activity. The free energy diagrams at the Ni site and Cu doped at the Ni site are presented in Figure 7f, and the typical adsorption structures of the intermediates are summarized in Figure 7g. It can clearly be seen that Cu doping lowers the barrier for the oxidation of HMF and therefore enhances the activity of the material. The limiting potentials were calculated to be 1.49 and 1.17 V for Ni and Cu sites, respectively, which agree well with the experimental observations. On the other hand, the activity volcano (Figure 7e) suggests that the activity of the material should be enhanced as long as the doping weakens the binding of HMF* within a large region (light blue region in Figure 7e,h). We further calculated Δ*G*(HMF*) for Cd and Mg doped at the Ni site, for five metals doped at the Co site in model I, and for all of the sites on the surface metal atoms in model II (Figure 7i). As shown in Figure 7h, most of them (even the Mn site in model II) fall into the enhancing region, which agrees well with the experimental observation. Since the d-band center (ε_d_) is crucial for revealing the origin of the catalytic activities, we further explored the influence of each metal active site for HMF* adsorption. The ε_d_ of Cd, Zn, Cu, Ni, Co, and Mn in model II increased from −6.2 to −0.6 eV, while the corresponding free energy of HMF* decreased from −0.53 to −0.89 eV, indicating that the metals with higher ε_d_ exhibit stronger adsorption toward HMF* (Figure S38).

For the OER process, we performed a similar analysis. The OER mainly follows the pathway of H_2_O → OH* → O* → OOH* → O_2_ in an alkaline environment. We found that the Co site of the (Co, Ni)–OOH is more active than the Ni site; thus, the Co site was then doped by Mn, Cu, and Zn to establish the scaling relation (Figure S39) and the activity volcano (Figure 7j). Because the Co site is already very active for the OER,^49,50^ the Δ*G*(OH*) at the doped active site needs to fall into the region from −0.04 to 0.76 eV (light orange region in Figure 7j,k) to further enhance the OER activity. However, from Figure 7k, all of the Δ*G*(OH*) values of the active sites in models I and II fall out of the optimal region, suggesting that doping of these metals suppresses the OER activity. Therefore, the doping of the transition metals enhances the HMFOR and suppresses the OER, which leads to higher HMFOR activity and selectivity over the high-entropy hollow CC@LHA(7) electrocatalyst (Figure S40).

## Conclusions

In summary, we have synthesized a series of self-supported hollow high-entropy layered hydroxide arrays (HE-LHAs) on carbon cloth (CC) from the ZIF-L precursor by a facile metal–organic framework (MOF)-templated strategy. It is demonstrated that the septenary CC@CoNiMnCuZnCdMg-LHA electrocatalyst can accelerate 5-hydroxymethyl furfural (HMF) electrooxidation but suppress the oxygen evolution reaction (OER). By suppressing OER activity, it exhibits a low potential of 1.42 V_RHE_ for HMF electrooxidation but a high potential of 1.68 V_RHE_ for the OER to achieve 100 mA cm^–2^. Furthermore, it also exhibits good stability, while both the yield and FE of FDCA can remain close to 100% after 10 cycles. Finite element simulations show that the hollow leaf-like array morphology can induce a strong local electric field over all of the shell, which can favor the electrocatalytic process. *In situ* electrochemical impedance spectroscopy (EIS) and DFT calculations also reveal that the Co, Ni, Cu, Zn, Mn, Cd, and Mg metals in HE-LHAs accelerate the HMFOR but suppresses the OER.

## Supporting Information Available

Experimental section, computational methods, SEM images, TEM images, HPLC spectra, and additional data (PDF)
