# Supplementary material for: High-Entropy Engineering in Hollow Layered Hydroxide Arrays to Boost 5-Hydroxymethylfurfural Electrooxidation by Suppressing Oxygen Evolution
Source: ACS Cent Sci. 2024 Oct 3;10(10):1920–32. doi: 10.1021/acscentsci.4c01085 (PMC11503487; doi:10.1021/acscentsci.4c01085)
Supplement: Supplementary file 1 — oc4c01085_si_001.pdf [file oc4c01085_si_001.pdf]

Supporting Information

High-entropy engineering in hollow layered hydroxide arrays to boost 5-hydroxymethylfurfural electrooxidation by suppressing oxygen evolution

Yu Xin,[a] Hongchuan Fu,[a] Liyu Chen,[a] Yongfei Ji,[*b] Yingwei Li[a] and Kui Shen[*a]

[a] *Guangdong Provincial Key Lab of Green Chemical Product Technology, School of Chemistry and Chemical Engineering, South China University of Technology, Guangzhou 510640, China*

[b] *School of Chemistry and Chemical Engineering, Guangzhou University, Guangzhou, 510006, China*

* Corresponding authors.

E-mail: cekshen@scut.edu.cn (K. S.); yongfeiji2018@gzhu.edu.cn (Y. J.)

This file includes:

1. Chemicals.
2. Synthesis of various samples.
3. Characterizations.
4. Electrochemical measurements.
5. RHE calibration.
6. Product analysis.
7. Electricity consumption calculations
8. Finite element simulations.
9. Computational details.
10. Figures S1-S40.
11. Tables S1-S6.
12. References.

Chemicals

All the chemicals and solvents were obtained from commercial sources and used as received without further purification unless otherwise stated. Cobalt nitrate hexahydrate ($Co(NO_3)_2·6H_2O$, 99%, Aladdin Industrial Corporation), nickel nitrate hexahydrate ($Ni(NO_3)_2·6H_2O$, 99%, Aladdin Industrial Corporation), zinc nitrate hexahydrate ($Zn(NO_3)_2·6H_2O$, 99%, Aladdin Industrial Corporation), copper nitrate trihydrate ($Cu(NO_3)_2·3H_2O$, 99%, Aladdin Industrial Corporation), cadmium nitrate tetrahydrate ($Cd(NO_3)_2·4H_2O$, 99%, Aladdin Industrial Corporation), manganese nitrate tetrahydrate ($Mn(NO_3)_2·4H_2O$, 98%, Adamas Industrial Corporation), magnesium nitrate hexahydrate ($Mg(NO_3)_2·6H_2O$, 98%, Guangzhou Tongyuan Sci-Tech Co., Ltd), 2-methylimidazole ($C_4H_6N_2$, 98%, Aladdin Industrial Corporation), 5-hydroxymethyl-2-furaldehyde (HMF, $C_6H_6O_3$, 99%, Macklin Biochemical Technology Co., Ltd.), 5-hydroxymethyl-2-furancarboxylic acid (HMFCA, $C_6H_6O_4$, 97%, Aladdin Industrial Corporation), 2,5-diformylfuran (DFF, $C_6H_5O_3$, 99%, Aladdin Industrial Corporation), 5-formyl-2-furancarboxylic acid (FFCA, $C_6H_5O_4$, 99%, Aladdin Industrial Corporation), 2,5-furandicarboxylic acid (FDCA, $C_6H_4O_5$, 99%, Aladdin Industrial Corporation), potassium hydroxide (KOH, 95 %, Aladdin Industrial Corporation), ammonium formate (99%, Aladdin Industrial Corporation), methanol (C_2H_6O, 99%, Guangdong Guanghua Sci-Tech Co., Ltd), ethanol (C_2H_6O, 99%, Guangdong Guanghua Sci-Tech Co., Ltd) were used. Commercial carbon cloth (CC) was obtained from the Zebo Electrochemical Materials Co., Ltd. The purification of water is carried on the pure water preparation machine (UPTC-20, Shanghai Lichen Bangxi Instrument Technology Co., Ltd) by passing through ion-exchange column to get distilled water with a resistance of 18.2 MΩ cm^{-1}.

Synthesis of CC@ZIF-L

A piece of carbon cloth (CC) with a size of 30 × 40 × 0.36 mm^3 was firstly washed with acetone ultrasonically to remove the oily pollutant. Then the CC was immersed into a mixed acid solution containing concentrated nitric acid (HNO_3, 10 mL) and concentrated sulfuric acid (H_2SO_4, 30 mL) for 24 h. The as-obtained CC was washed with deionized water and ethanol several times to ensure that the surface of the CC was well cleaned before further use. After

that, the CC substrate supported by stent was immersed in distilled water (H_2O, 80 mL) containing 2-methylimidazole (1.313 g, 16 mmol) and $Co(NO_3)_2{\cdot}6H_2O$ (0.583 g, 2 mmol) for 4 h at room temperature (25 ℃) to obtained ZIF-L on CC (denoted as CC@ZIF-L). Furthermore, the as-obtained CC@ZIF-L sample with a light purple color was washed with H_2O for several times until the water turned colorless before further use and further dried in an oven at 60°C for 24 h.

Synthesis of CC@LHA(2)

A piece of CC@ZIF-L was immersed in 8 mM $Ni(NO_3)_2$ ethanol solution for 60 min at 30 ℃. Then, the product was taken out, washed with ethanol and finally dried at 60 °C for 24 h to obtain the final CC@LHA(2).

Synthesis of CC@LHA(4)

A piece of CC@ZIF-L was immersed in ethanol solution with $Ni(NO_3)_2{\cdot}6H_2O$ (8 mM), $Zn(NO_3)_2{\cdot}6H_2O$ (8 mM), $Cu(NO_3)_2{\cdot}3H_2O$ (8 mM) for 60 min at 30 ℃. Then, the product was taken out, washed with ethanol and finally dried at 60 °C for 24 h to obtain the final CC@LHA(4).

Synthesis of CC@LHA(5)

A piece of CC@ZIF-L was immersed in ethanol solution with $Ni(NO_3)_2{\cdot}6H_2O$ (8 mM), $Zn(NO_3)_2{\cdot}6H_2O$ (8 mM), $Cu(NO_3)_2{\cdot}3H_2O$ (8 mM), $Mn(NO_3)_2{\cdot}4H_2O$ (8 mM) for 60 min at 30 ℃. Then, the product was taken out, washed with ethanol and finally dried at 60 °C for 24 h to obtain the final CC@LHA(5).

Synthesis of CC@LHA(6)

A piece of CC@ZIF-L was immersed in ethanol solution with $Ni(NO_3)_2{\cdot}6H_2O$ (8 mM), $Zn(NO_3)_2{\cdot}6H_2O$ (8 mM), $Cu(NO_3)_2{\cdot}3H_2O$ (8 mM), $Cd(NO_3)_2{\cdot}4H_2O$ (8 mM), $Mn(NO_3)_2{\cdot}4H_2O$ (8 mM) for 60 min at 30 ℃. Then, the product was taken out, washed with ethanol and finally dried at 60 °C for 24 h to obtain the final CC@LHA(6).

Synthesis of CC@LHA(7)

A piece of CC@ZIF-L was immersed in ethanol solution with $Ni(NO_3)_2·6H_2O$ (8 mM), $Zn(NO_3)_2·6H_2O$ (8 mM), $Cu(NO_3)_2·3H_2O$ (8 mM), $Cd(NO_3)_2·4H_2O$ (8 mM), $Mn(NO_3)_2·4H_2O$ (8 mM), $Mg(NO_3)_2·6H_2O$ (8 mM) for 60 min at 30 ℃. Then, the product was taken out, washed with ethanol and finally dried at 60 °C for 24 h to obtain the final CC@LHA(7).

Characterization

SEM was conducted on HITACHI SU8220 scanning electron microscope. TEM with EDS analysis (Bruker Xflash 5030T) and high angle annular dark field scanning transmission electron microscopy (STEM) were conducted on a high-resolution transmission electron microscope (JEOL, JEM-2100 F) operated at 200 kV. The X-ray diffraction (XRD) analysis was performed with a Bruker D8 Advance X-ray diffractometer (Bruker Co., Germany) with a nickel-filtered Cu Kα radiation source (30 kV/160 mA, λ= 0.1543 nm). The diffraction peaks were attributed to the corresponding crystalline phases by comparing published references and the powder diffraction files from the Joint Committee on Power Diffraction Standards (JCPDS). X-ray photoelectron spectroscopy (XPS) was taken on Nexsa Thermo ESCALAB 250XI with an Al Kα X-ray source (Mono AlKa, 1486.6 eV). The X-ray absorption spectroscopy (XAS) tests were recorded on the Rapid XAFS 2 M (Anhui Absorption Spectroscopy Analysis Instrument Co., Ltd.) by transmission (or fluorescence) mode at 20 kV and 20 mA, and the Si (533) spherically bent crystal analyzer with a radius of curvature of 500 mm was used. The contents of different metal elements were measured by Flame Atomic Absorption Spectrophotometer (Agilent 240FS AA). The ^1H nuclear magnetic resonance (NMR) were performed on Bruker Advance/AV 500 MHz. The Fourier transform infrared (FT-IR) spectrometer were performed on Bruker Tensor.

Electrochemical measurements

The electrochemical measurements were performed in a three electrodes system by using CHI 760E electrochemical workstation (Shanghai Chenhua Co., Ltd., China) with our samples (exposed geometric area of 1 × 1 cm^2), Pt sheet and saturated Hg/HgO electrodes as working, counter, and reference electrodes, respectively. The potentials were calibrated to RHE

according to the following equation: E(RHE) = E(Hg/HgO) + 0.921. Cyclic voltammetry (CV) and linear sweep voltammetry (LSV) experiments were scanned in Ar-saturated 1 M KOH electrolyte at a rate of 5 mV s^{-1} to minimize the effect of capacitance. The LSV curves of HER are corrected with 90% iR correction to correct the solution resistance caused ohmic potential drop. The corresponding Tafel plots were calculated by the equation of $\eta = b \log j + a$. Operando electrochemical impedance spectroscopy (EIS) was performed with a frequency range from 0.1 Hz to 10 kHz and AC amplitude of 5 mV. *In situ* Raman spectroscopy was performed on LabRAM HR (Horiba Jobin-Yvon, France) using a 532 nm laser, with a three-electrode C031 cell (GaossUnion) for *in situ* measurements. All the electrochemical curves were collected until the test results kept a stable state at room temperature.

RHE calibration

We used saturated Hg/HgO (GaossUnion) as a reference electrode in alkaline electrochemical test in the absence of specific instructions. The potential was referred to reversible hydrogen electrode (RHE) by calibrating the reference electrode with Pt wire electrode (GaossUnion) and Pt nets electrode (GaossUnion) were employed as the working and counter electrode in a sealed standard three-electrode. All the potentials reported in our manuscript were calibrated to RHE by the following equation: E(REH) = E(SCE) + 0.921 V.

Product analysis

The concentrations of the organics during HMF electrooxidation process were determined by HPLC (Agilent Technologies 1260 Infinity II) with an ultraviolet-visible detector (set to 265 nm) and Shim-pack GWS 5 μm C18 column (4.6 mm×150 mm). The HPLC eluent consisted of 70% (v/v) 5 mM ammonium formate (FA) aqueous solution and 30% (v/v) methanol have a total flow of 0.6 mL min^{-1}, and each separation lasts for 6 min. Specifically, we took 10 μL of electrolyte sample from the system, diluted it by using 490 μL ultrapure water and analyzed the diluted solution by HPLC. The standard curve was obtained by using standard pure solutions of organics. The HMF conversion, FDCA yield, and Faradaic efficiency were calculated by using the equations of (1), (2), and (3), respectively.

$$\text{HMF conversion (\%)} = \frac{HMF\ \ consumed\ \ (mol)}{HMF\ \ added\ \ (mol)} \times 100 \qquad (1)$$

$$\text{FDCA yield (\%)} = \frac{FDCA\ formed\ (mol)}{HMF\ added\ (mol)} \times 100 \qquad (2)$$

$$\text{Faradaic efficiency (\%)} = \frac{FDCA\ formed\ (mol)}{Q/(6 \times F)} \times 100 \qquad (3)$$

here Q is the transferred charge, and F is the Faraday constant (96485 C mol^{-1}).

Electricity consumption calculations

For two-electrode electrolysis in 1 M KOH electrolyte containing 20 mM HMF, CC@LHA(7) and Pt foil were employed as an anode and a cathode, receptively. The electricity consumption of the HMFOR-coupled H_2 production system was calculated by the following equation: $W = (n \times F \times U) / (3.6 \times 10^6 \times V_m \times 10^{-3})$

W: the electricity consumption of producing 1 stere H_2, kWh per stere H_2.

n: the number of electrons transferred in H_2 production, which is 2.

F: Faraday constant, which is 96485 C mol^{-1}.

U: the applied voltage.

V_m: molar volume of gas at room temperature of 25°C and normal pressure of 101 kPa by van der Waals equation, which is 24.5 L mol^{-1}.

Finite element simulations (FES)

Electric field around electrode were simulated using the COMSOL Multiphysics finite-element-based solver. The "Electric Currents" module was used to simulate the electric field and potentials of 1.3 V was applied to the bottom of the array. The terminal was prescribed to the far side of the electrolyte with 0 V. Electric insulation was applied to the remaining electrolyte sides, and an initial value of 0 V was set everywhere.

Computational details

We have performed first-principles calculations with density functional theory implanted in the Vienna ab initio Simulation Package[1-3]. The Perdew–Burke–Ernzerhof[4] functional was applied with the electron-ion interaction described by the PAW pseudopotential[5]. The GGA+U[6] method was adopted with an effective U-J value of 3.0 eV[7] and 5.5 eV[8] to Co and Ni, and a value of 4.0 eV to Mn and Cu d-electrons[9]. A cutoff energy of 460 eV was applied for the plane-wave basis set. The edge sites of the (Co, Ni)-OOH were investigated because it was found

responsible for oxygen evolution reactions (OER)[10-12]. For OER, a four-layer slab with 16 transition metal ions was applied, while for the oxidation of HMF, a four-layer slab with 32 transition metal ions was applied with a vacuum layer of 14 Å. Two models were applied to understand the effect of the doping of transition metals. In model I, a single transition metal atom was doped into the Ni or Co site of (Co, Ni)-OOH. In Model II, five transition metal atoms (Mn, Cu, Zn, Cd, Mg) are doped into the first layer of the (Co, Ni)-OOH slab. The Brillouin zone was sampled by a Γ-point centered (1x2x1) Monkhorst[13] grid of k-points for the smaller supercell, and the Γ-point only for the larger supercell because of the large computational cost. The implicit solvation model was applied via VASPsol[14], and the dispersive interaction was included via the DFT+D3 scheme[15,16]. With the atoms in the bottom two layers fixed to their bulk positions, the structures were optimized until the maximum force on the atoms was smaller than 0.02 eV/Å.

The free energy surface was calculated based on the computational hydrogen electrode model (CHE)[17], in which the H^+ transfer is always coupled with the electron transfer so that the reaction energies can be related to the energy of H_2(g) and the potential U. Under this model, the HMFOR intermediates are either oxidized by dehydrogenation or by adding of hydroxyl group. The corresponding intermediates formed are denoted as Int-Hx (x= C, O) or Int+OH. The free energy corrections were included in the harmonic approximation[18]. The gas-phase molecules were considered ideal gas.[18] For OER, the overpotential for each step is defined as $\eta = \Delta G/e - 1.23$ V.

The Bader charges of the metal ions in LHA(2), LHA(6) and LHA(7) were also calculated. We used a 3x3 supercell with two Cu, two Zn, one Co, Ni, Mn, Cd, Mg ions to model LHA(7) because the ratio of Cu and Zn is much higher than other metals. The Mg atom is then replaced by a Zn to simulate LHA(6), while for LHA(2) a 2x2 supercell was used.

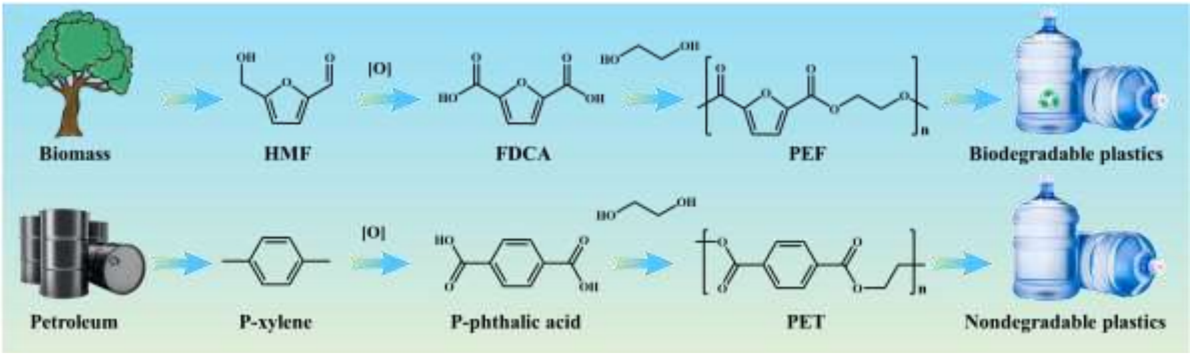

Figure S1 The synthetic route comparation of biomass-derived biodegradable PEF plastics and petroleum-derived PET plastics

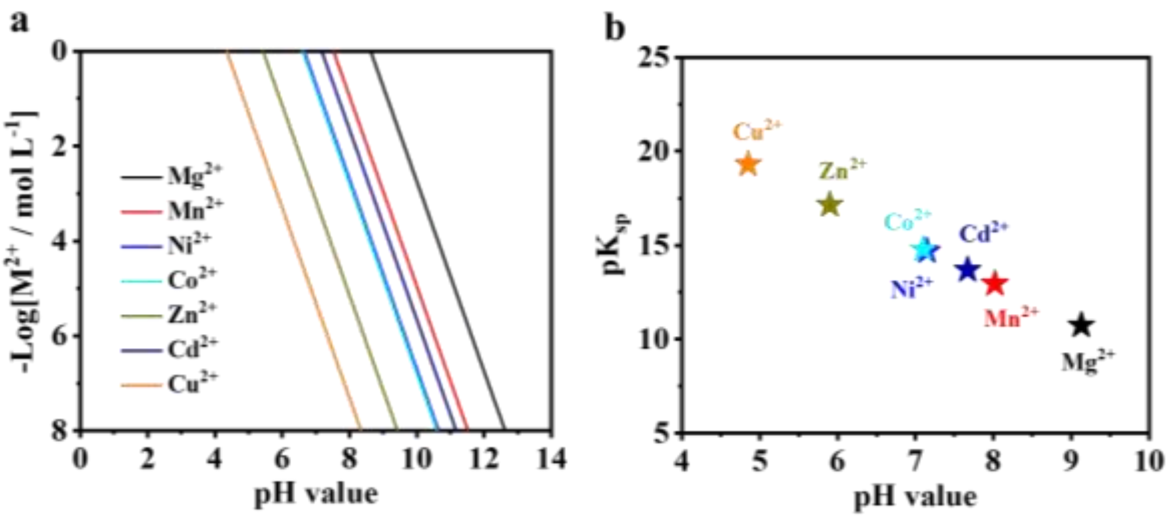

Figure S2. (a) The negative logarithm of the ion concentration vs. the pH value for different ions and (b) the corresponding pK_{sp} beginning to precipitate at 0.1 mol L^{-1}.

Formula 3 can be obtained by incorporating **Formula 1** into **Formula 2**.

Formula 1: $K_{sp} = C(M^{2+})^m \cdot C(OH^-)^n$

Formula 2: $pK_{sp} = -\log(K_{sp})$

Formula 3: $pK_{sp} = -\log(K_{sp}) = -\log[C(M^{2+})] - 2\log[C(OH^-)] = -\log[C(M^{2+})] + 2pH$

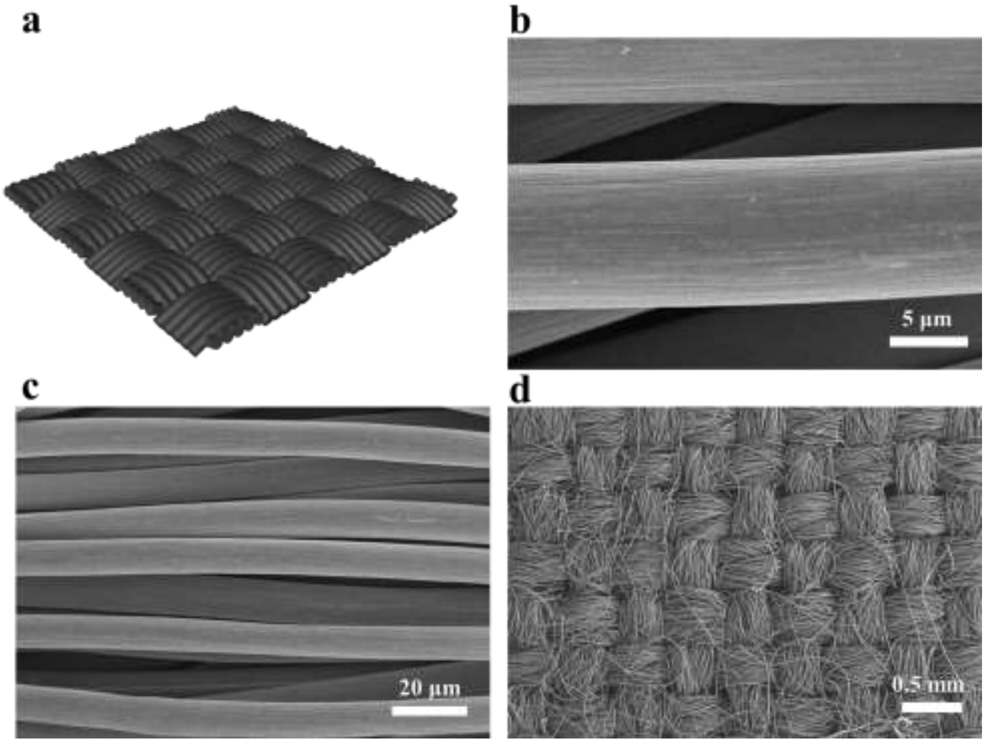

Figure S3. (a) The schematic model, and (b-d) low- and high-magnification SEM images of carbon cloth (CC).

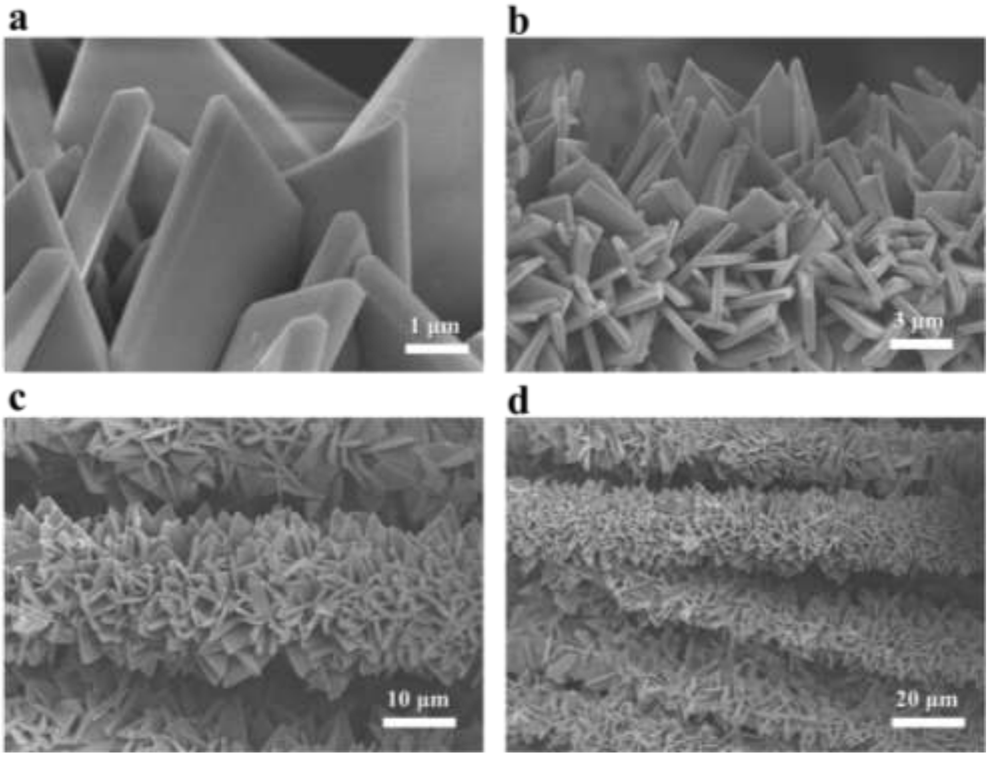

Figure S4. (a-d) Low- and high-magnification SEM images of CC@ZIF-L.

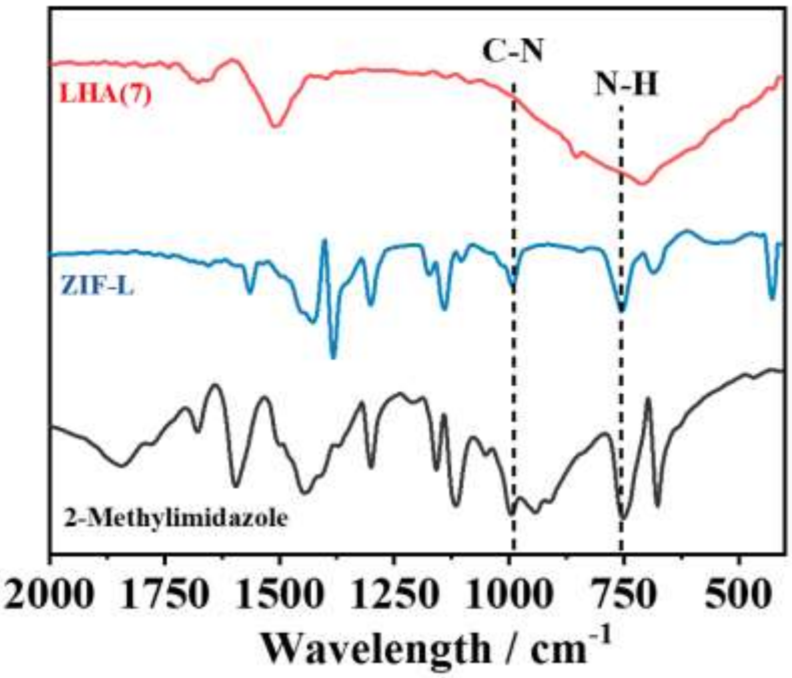

Figure S5. The FTIR spectra of CC@LHA(7), CC@ZIF-L and 2-methylimidazole.

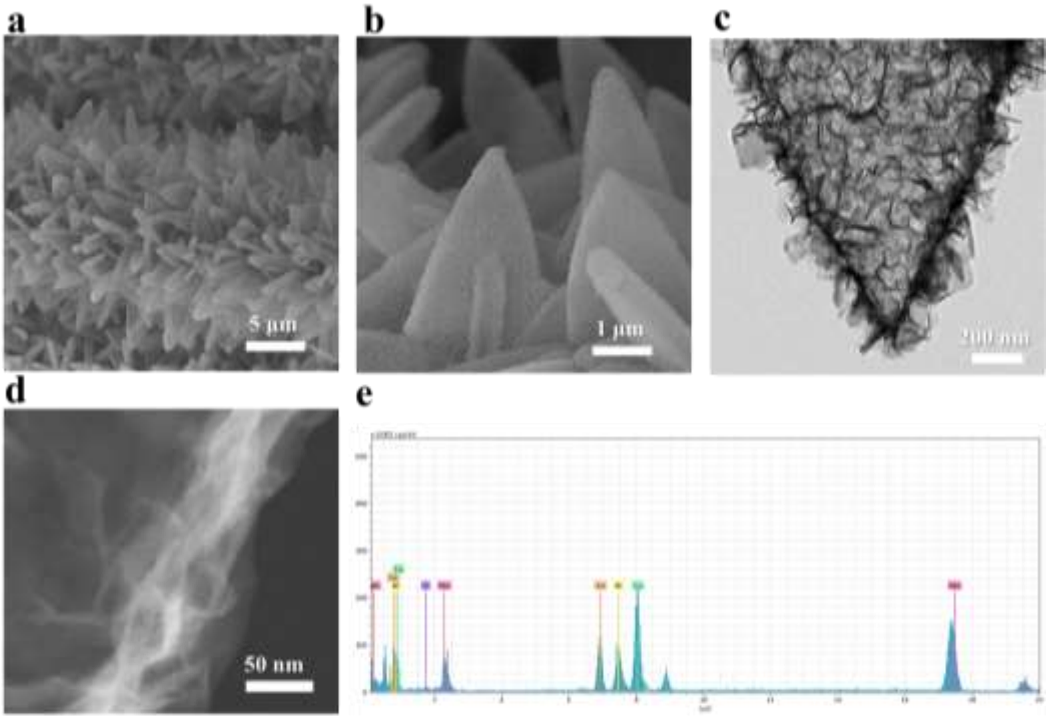

Figure S6. (a, b) Low- and high-magnification SEM, (c) TEM, (d) HAADF-STEM images and (e) the corresponding elemental spectrum of CC@LHA(2).

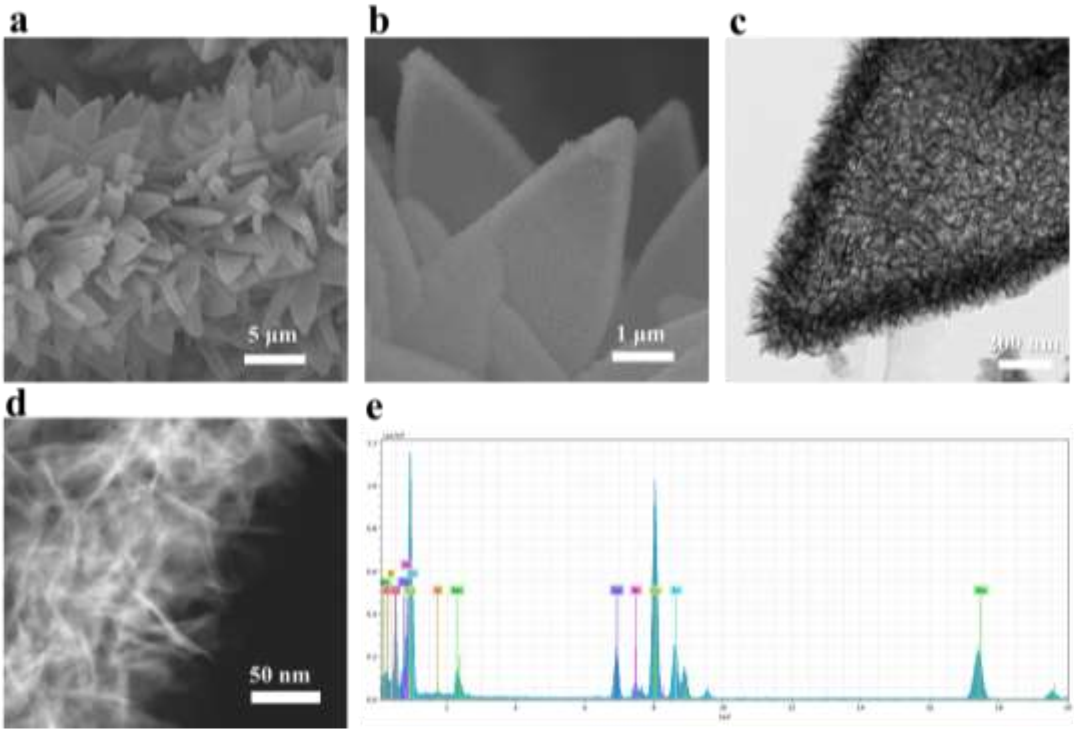

Figure S7. (a, b) Low- and high-magnification SEM, (c) TEM, (d) HAADF-STEM images and (e) the corresponding elemental spectrum of CC@LHA(4).

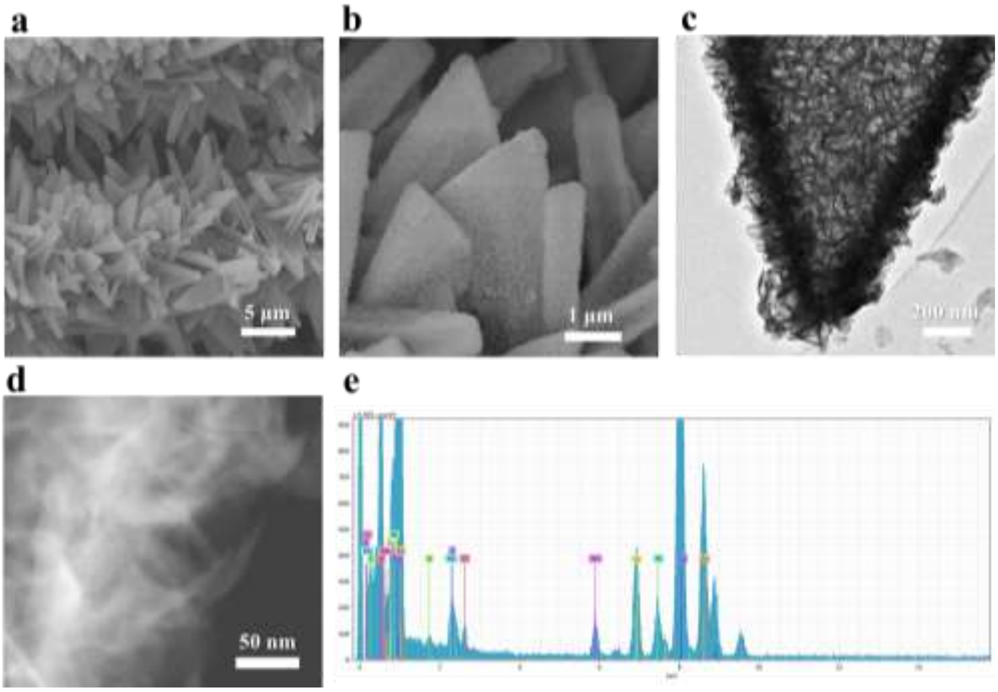

Figure S8. (a, b) Low- and high-magnification SEM, (c) TEM, (d) HAADF-STEM images and (e) the corresponding elemental spectrum of CC@LHA(5).

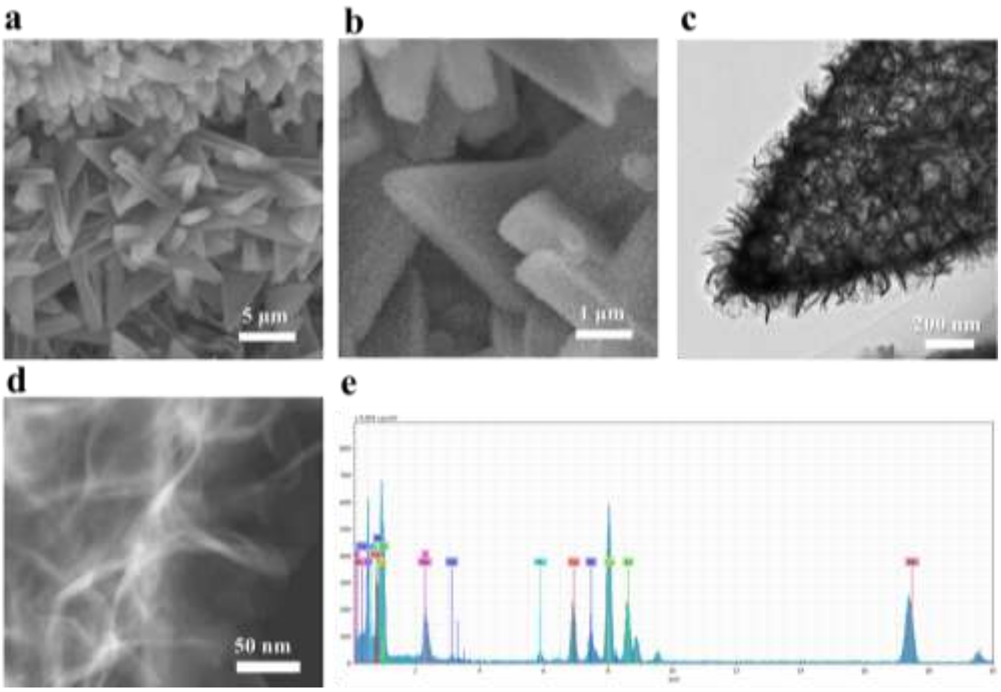

Figure S9. (a, b) Low- and high-magnification SEM, (c) TEM, (d) HAADF-STEM images and (e) the corresponding elemental spectrum of CC@LHA(6).

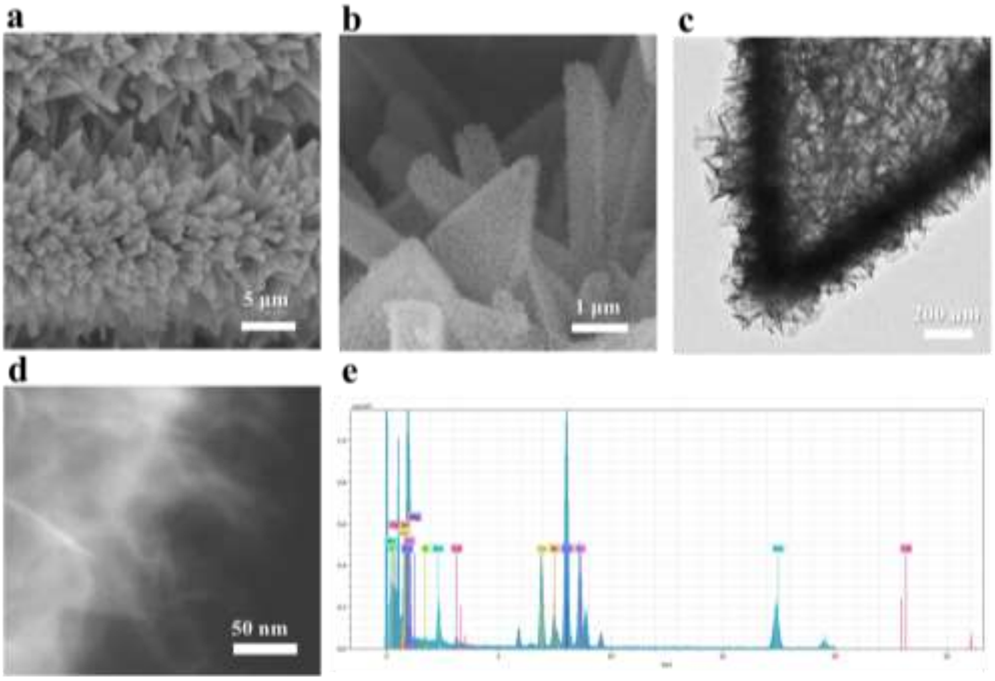

Figure S10. (a, b) Low- and high-magnification SEM, (c) TEM, (d) HAADF-STEM images and (e) the corresponding elemental spectrum of CC@LHA(7).

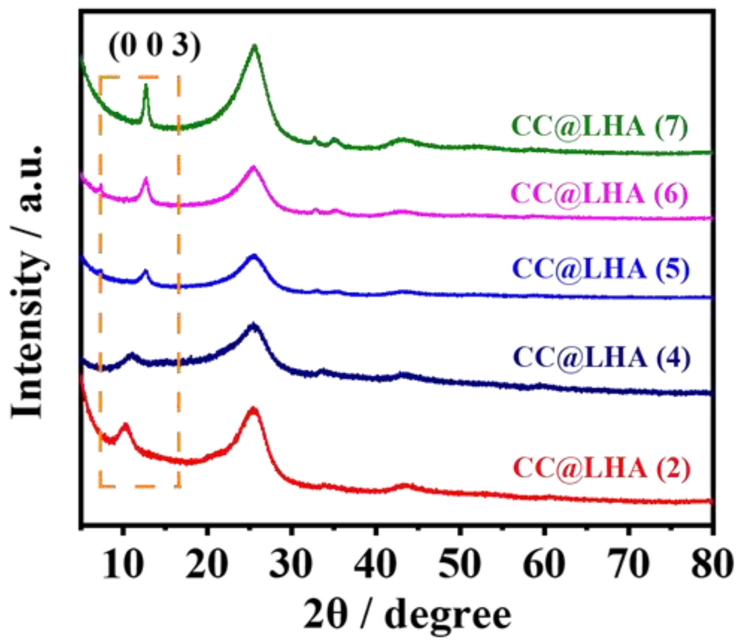

Figure S11. XRD patterns of CC@ZIF-L and CC@LHA(n).

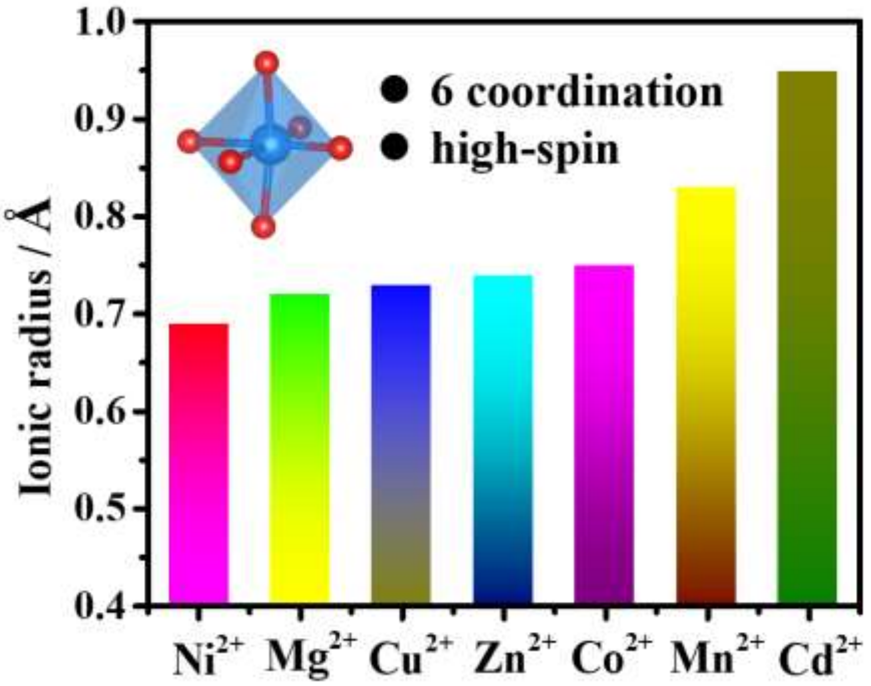

Figure S12. The ionic radius of different metallic elements with high-spin 6 coordination.

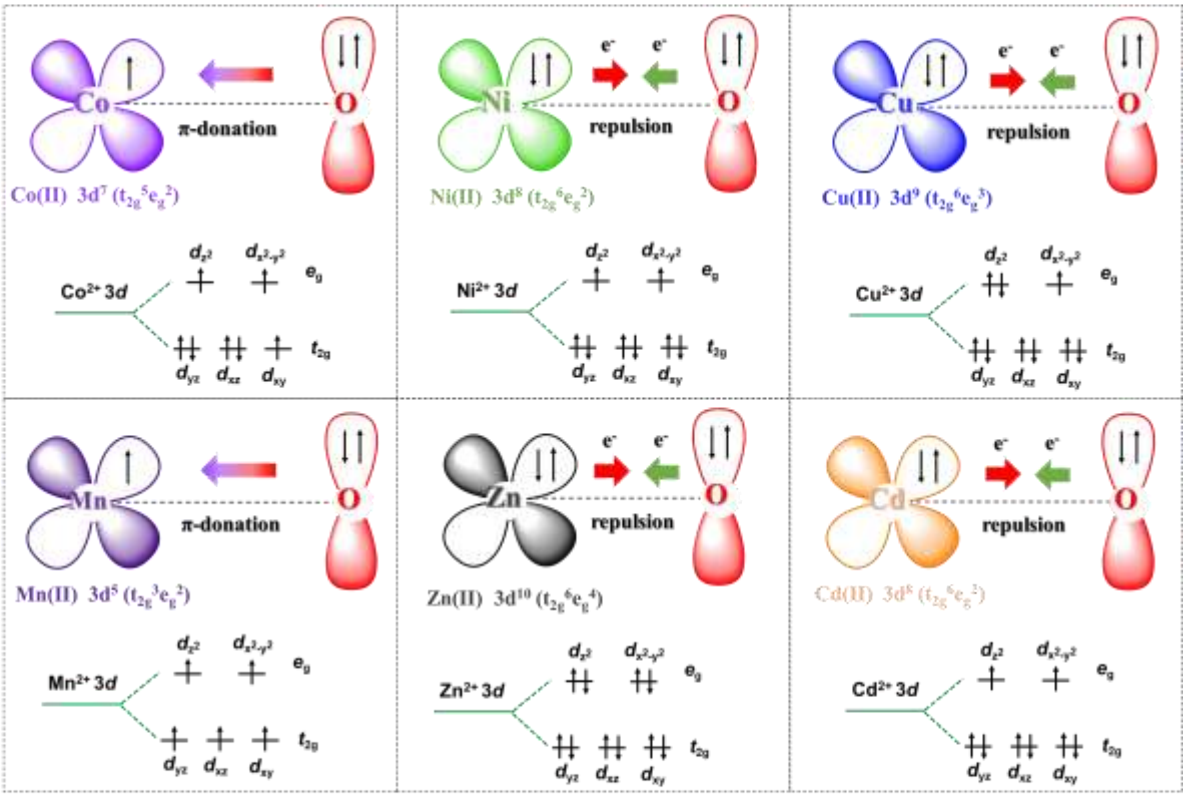

Figure S13. Schematic representations of the electronic coupling between O and Co, Ni, Cu, Mn, Zn, and Cd in LHAs structure.

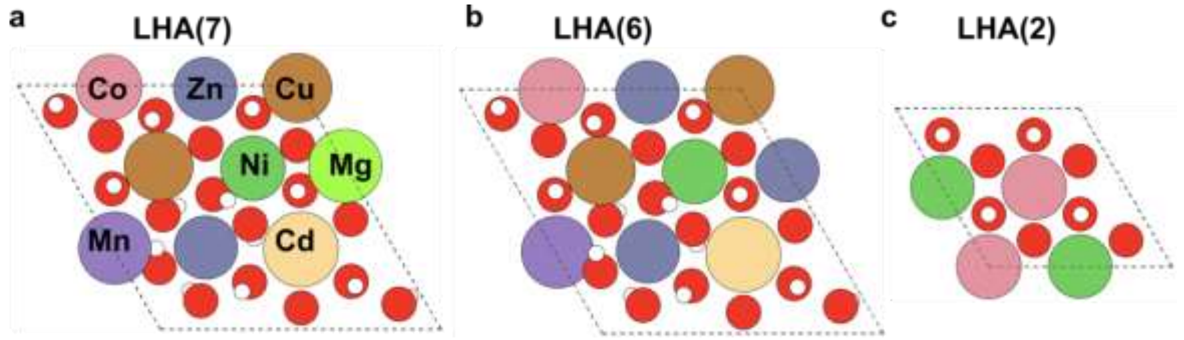

Figure S14. The Bader charges of the metal ions in (a) LHA(7), (b) LHA(6) and (c) LHA(2).

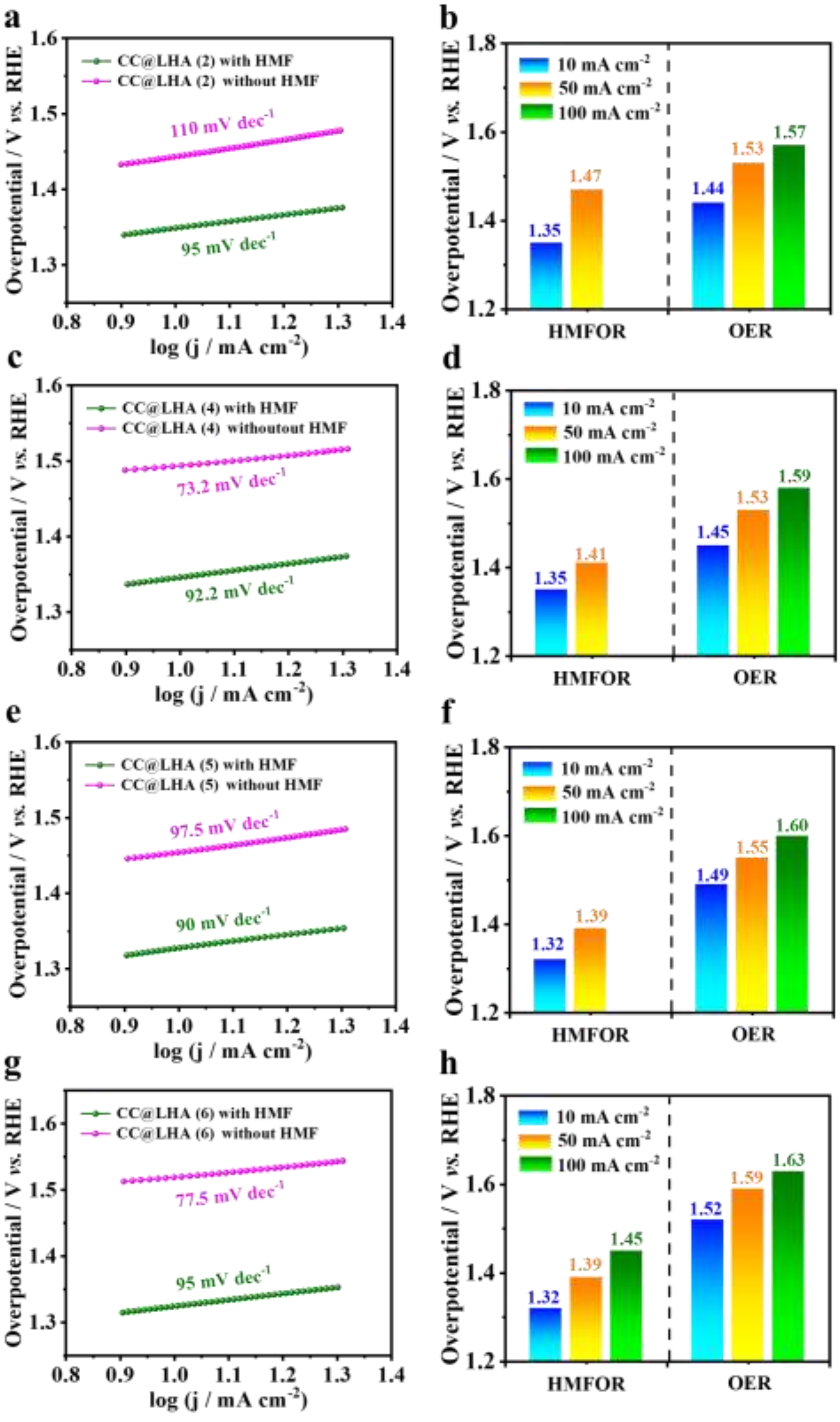

Figure S15. Tafel slopes and overpotentials of (a, b) CC@LHA(2), (c, d) CC@LHA(4), (e, f) CC@LHA(5), and (g, h) CC@LHA(6).

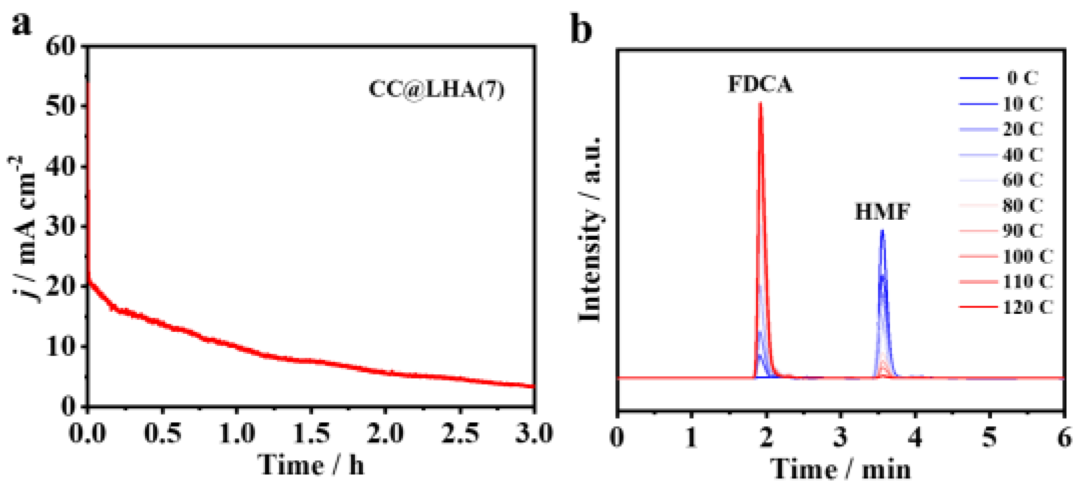

Figure S16. (a) I-t curve and (d) the corresponding HPLC chromatogram spectra for HMFOR over the CC@LHA(7) electrocatalyst.

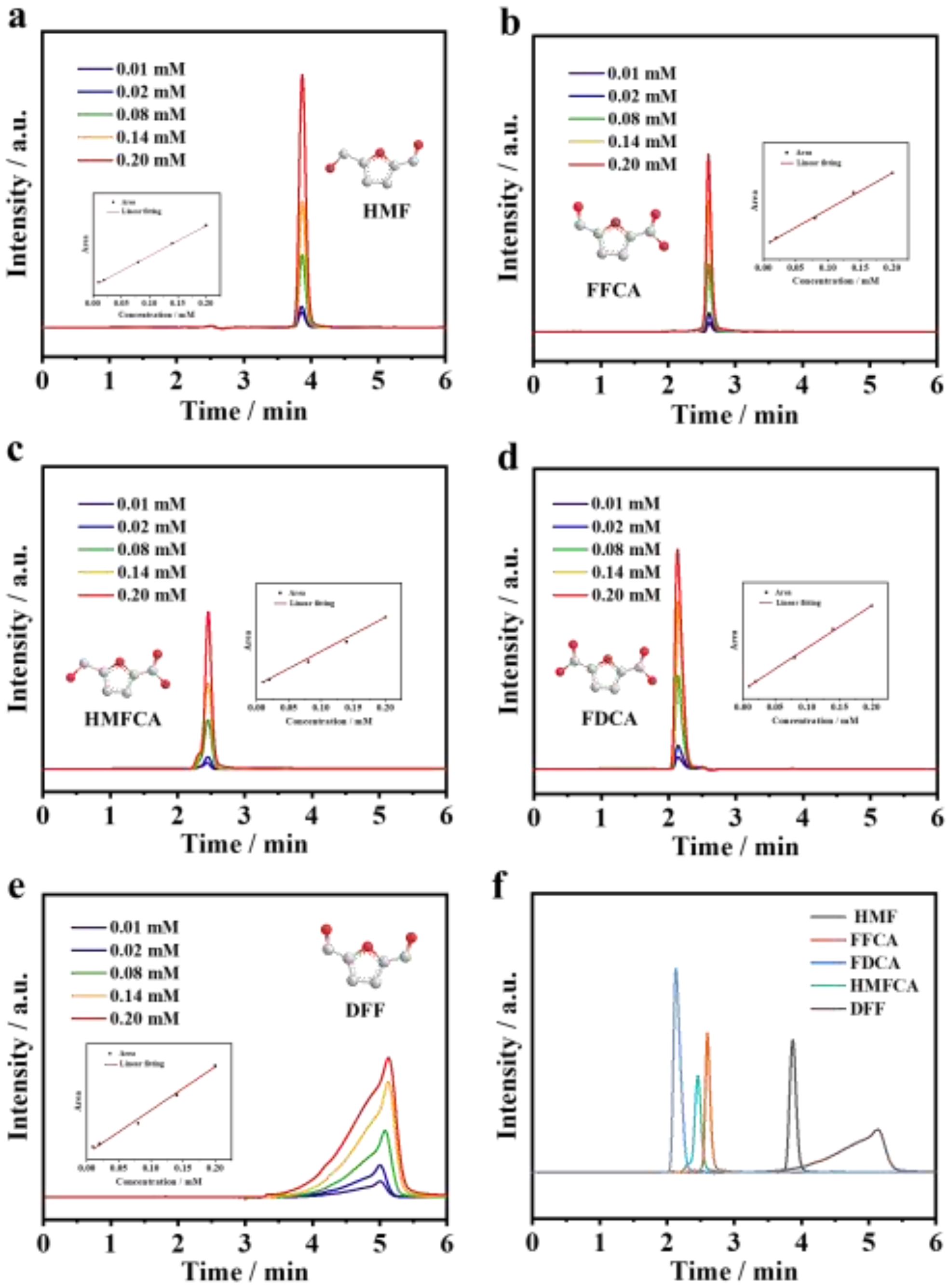

Figure S17. HPLC chromatogram spectra and the corresponding standard curves of (a) HMF, (b) FFCA, (c) HMFCA, (d) FDCA, and (e) DFF. (f) The elution curves of related standard mixtures.

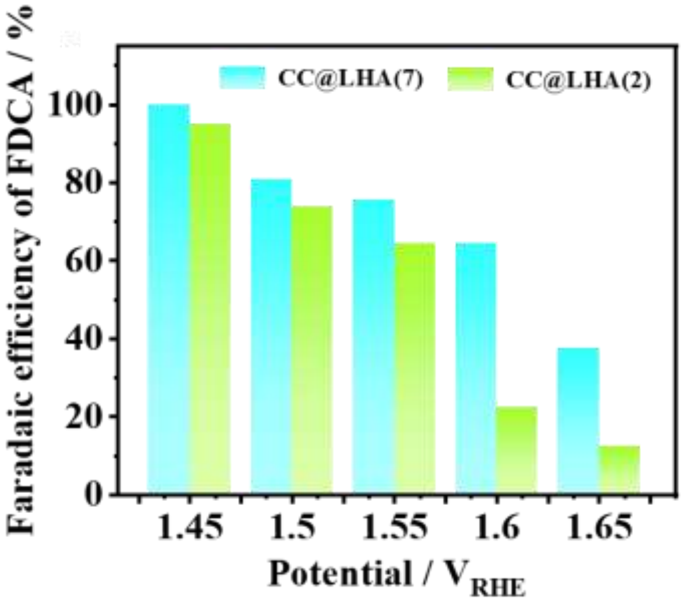

Figure S18. Faradaic efficiencies of FDCA at various potentials in 20 mM HMF solution over CC@LHA(2) and CC@LHA(7).

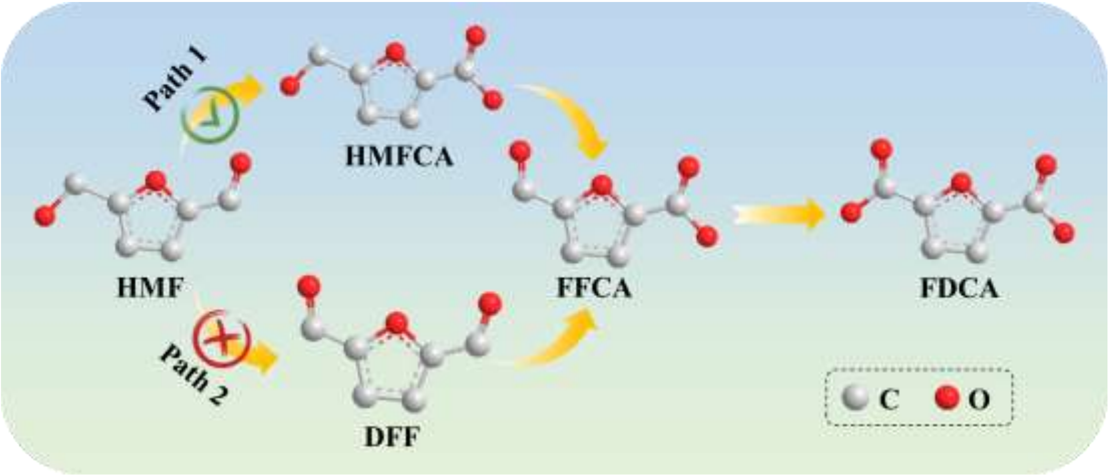

Figure S19. The proposed reaction pathways for HMFOR in alkaline electrolyte.

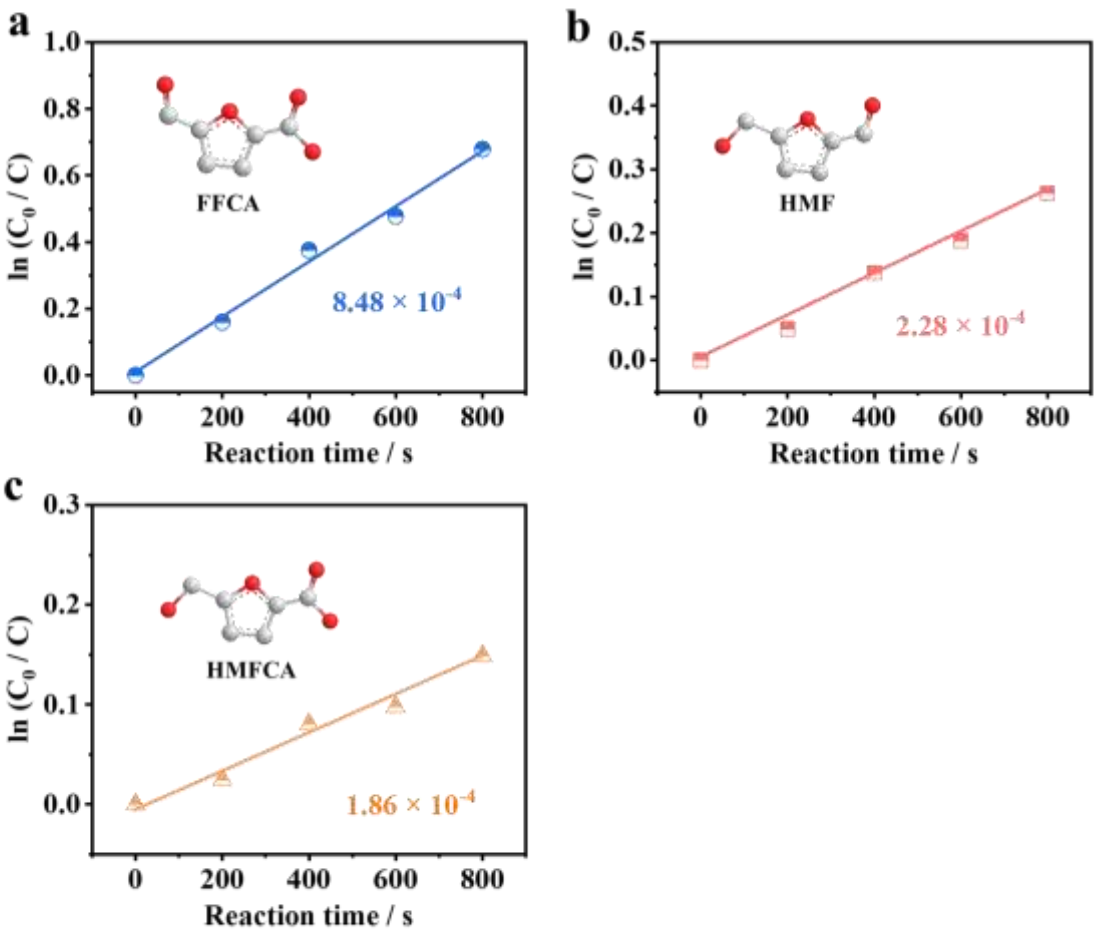

Figure S20. Reaction kinetic curves of (a) FFCA, (b) HMF, and (c) HMFCA electrooxidation based on $\ln(C_0/C)$ *vs.* the reaction time.

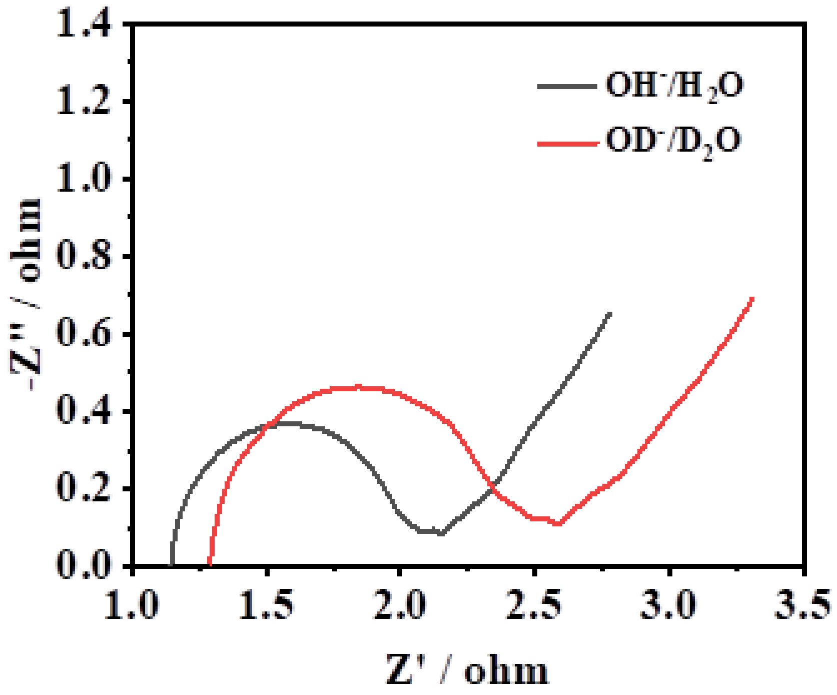

Figure S21. Nyquist plots of CC@LHA(7) in 0.1 M NaOH/H_2O and 0.1 M NaOD/D_2O electrolyte at 1.55 V_{RHE}.

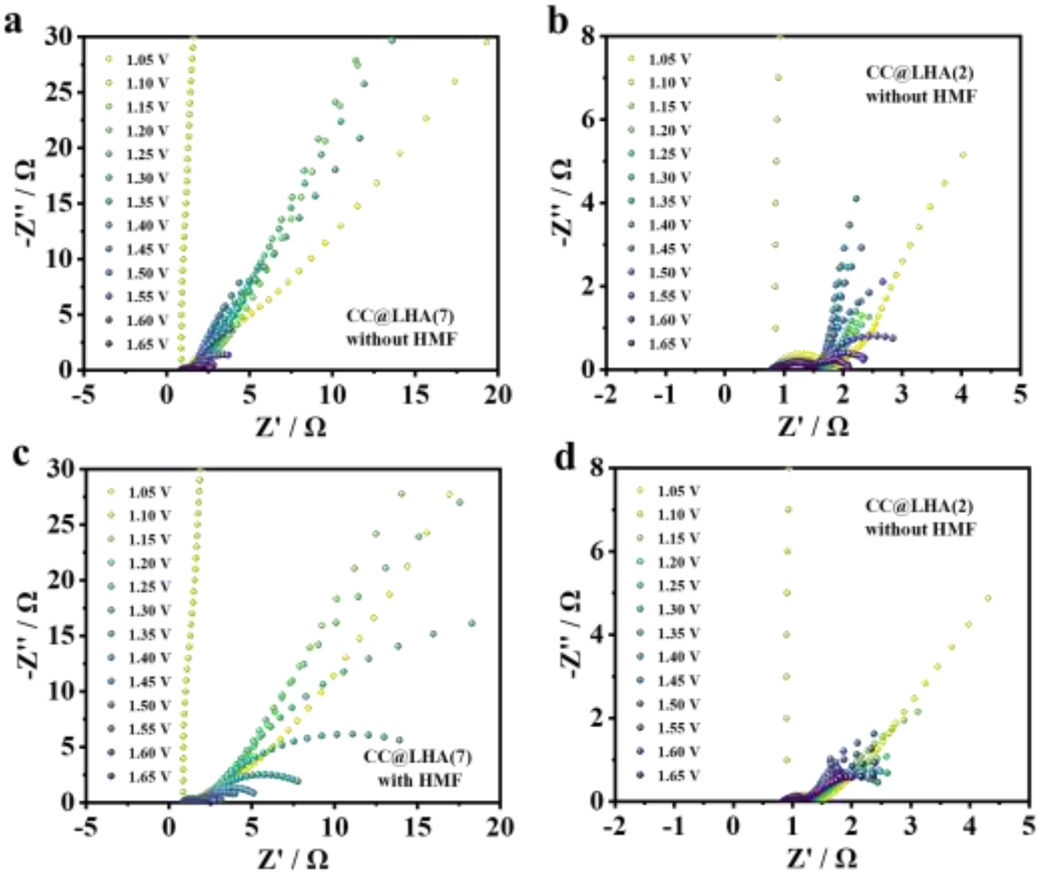

Figure S22. Nyquist plots of CC@LHA(2) and CC@LHA(7) in (a, b) 1.0 M KOH and (c, d) 1.0 M KOH with 20 mM HMF.

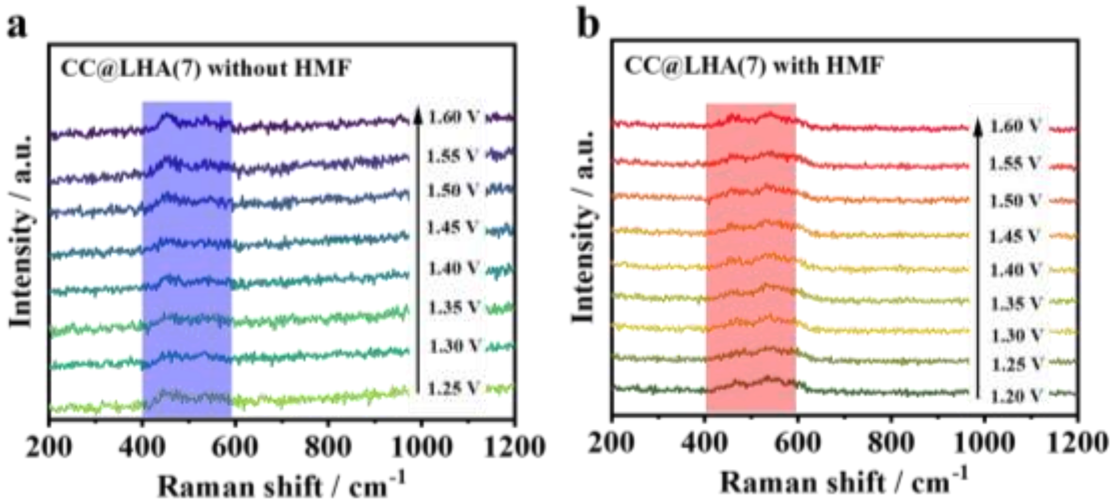

Figure S23. *In situ* Raman spectra for CC@LHA(7) in (a) 1 M KOH and (b) 1 M KOH with 20 mM HMF.

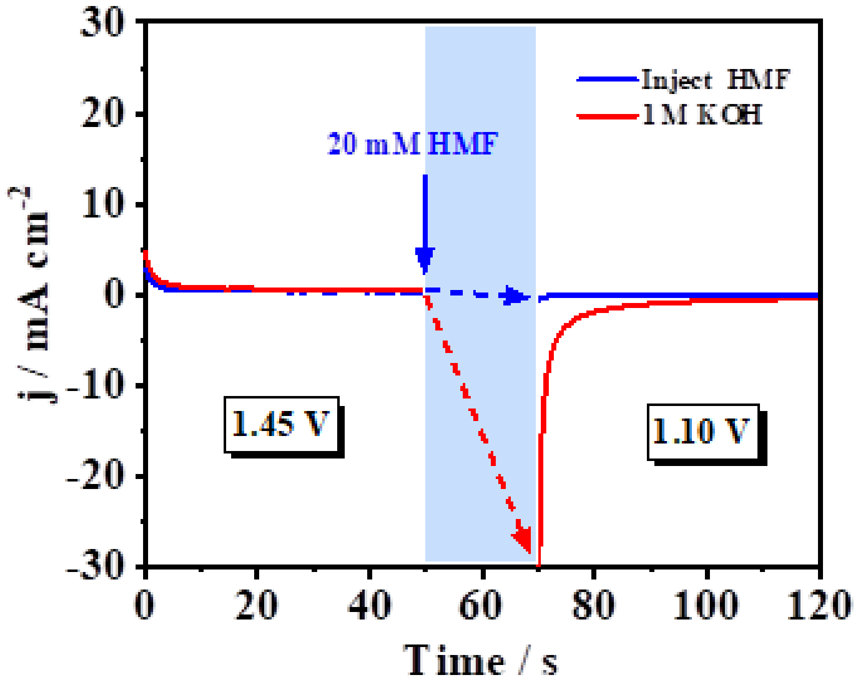

Figure S24. Multi-potential step curves of CC@LHA(7) with potential changed from 1.45 to 1.10 V_{RHE}.

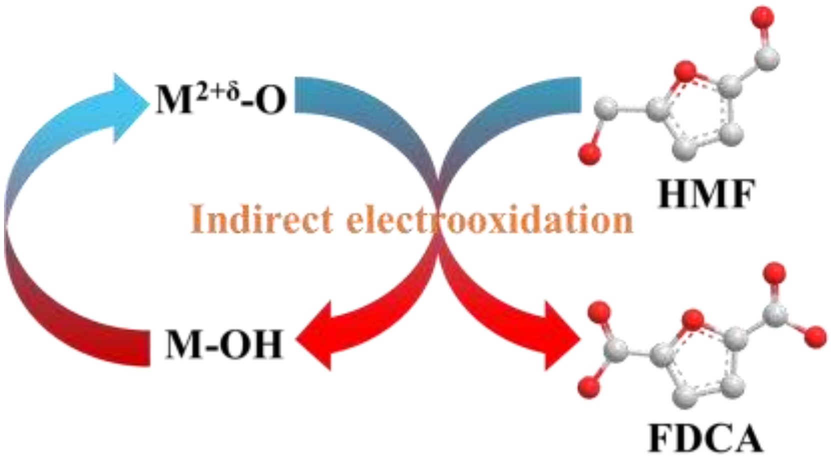

Figure S25. Schematic illustration of the indirect electrooxidation of HMF to FDCA.

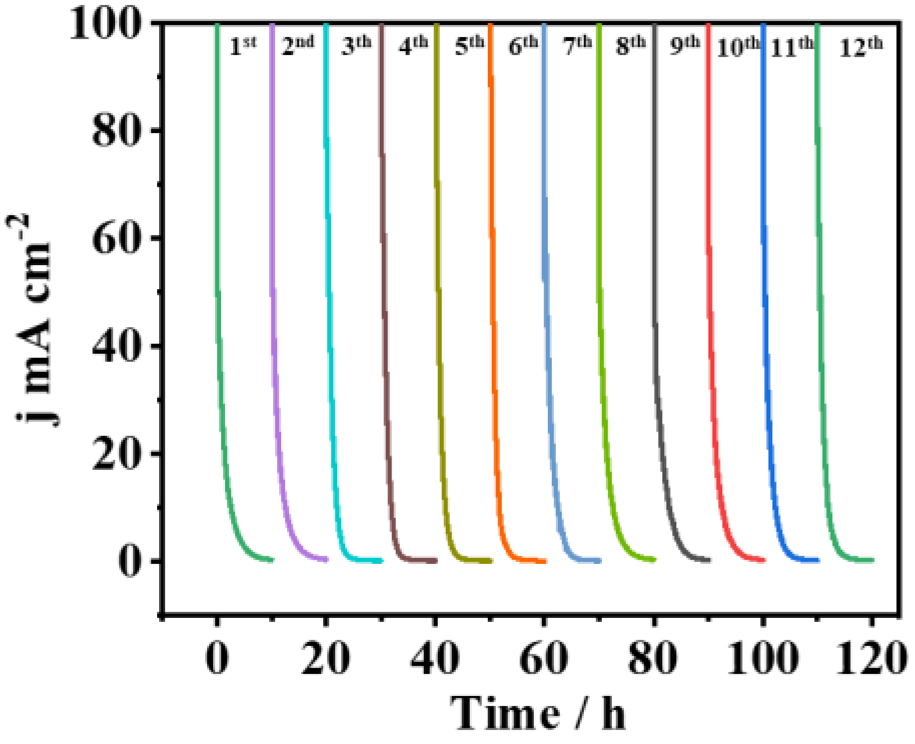

Figure S26. The long-term stability test for HMF electrooxidation over CC@LHA(7).

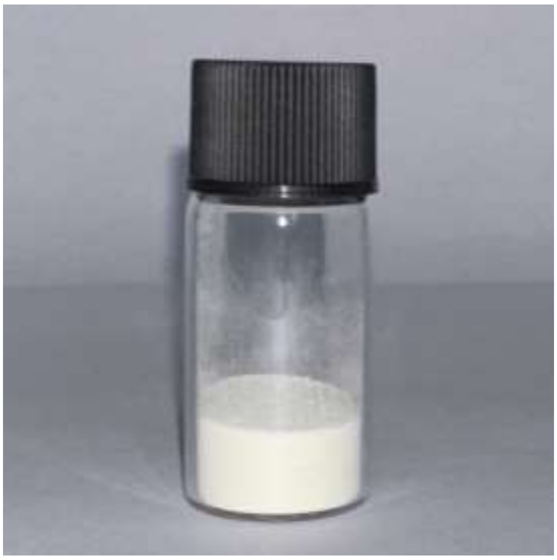

Figure S27 The gram-scale synthesized FDCA product collected after the continuous chronoamperometry test.

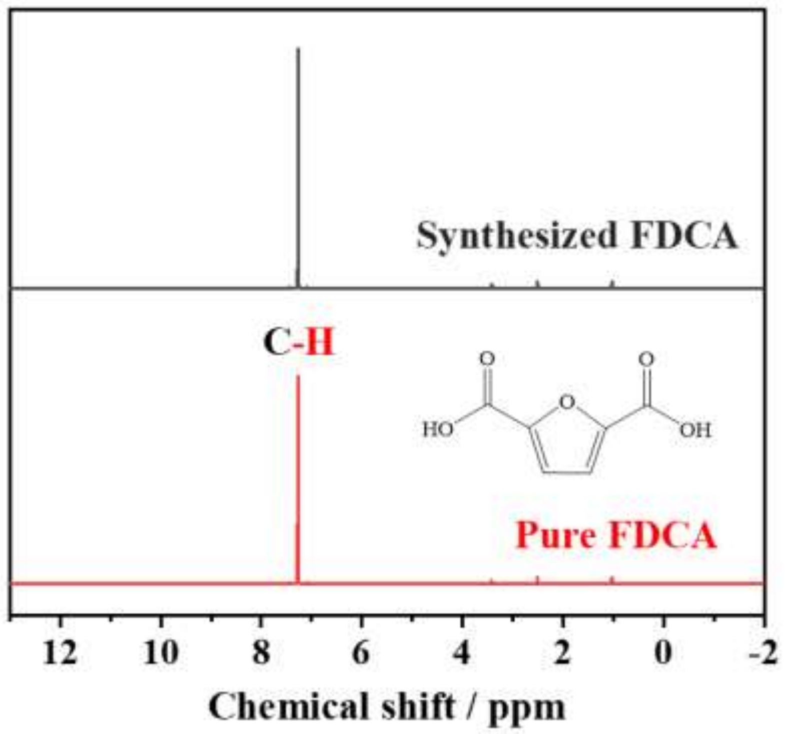

Figure S28 ^{1}H NMR spectra of the synthesized FDCA and pure FDCA (500 MHz, DMSO-d6, 298 K).

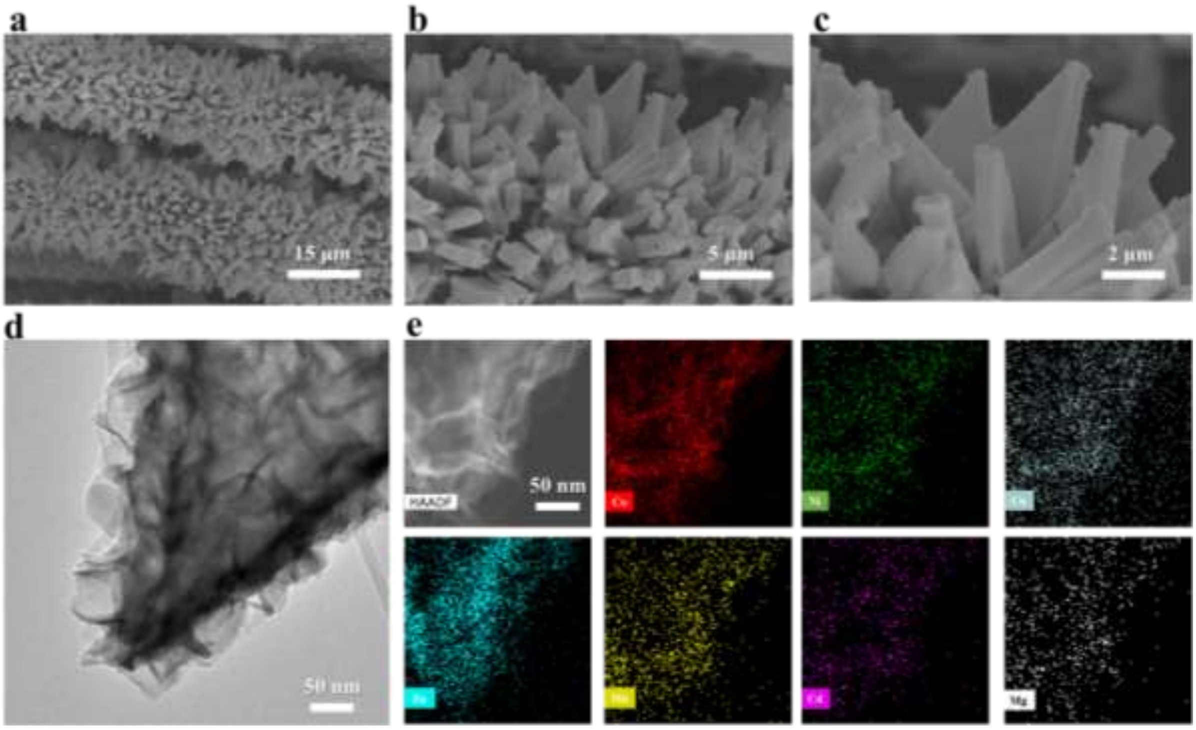

Figure S29. (a-c) Low- and high-magnification SEM, (d) TEM, (e) HAADF-STEM and the corresponding elemental mapping images of CC@LHA(7) after the electrocatalytic test.

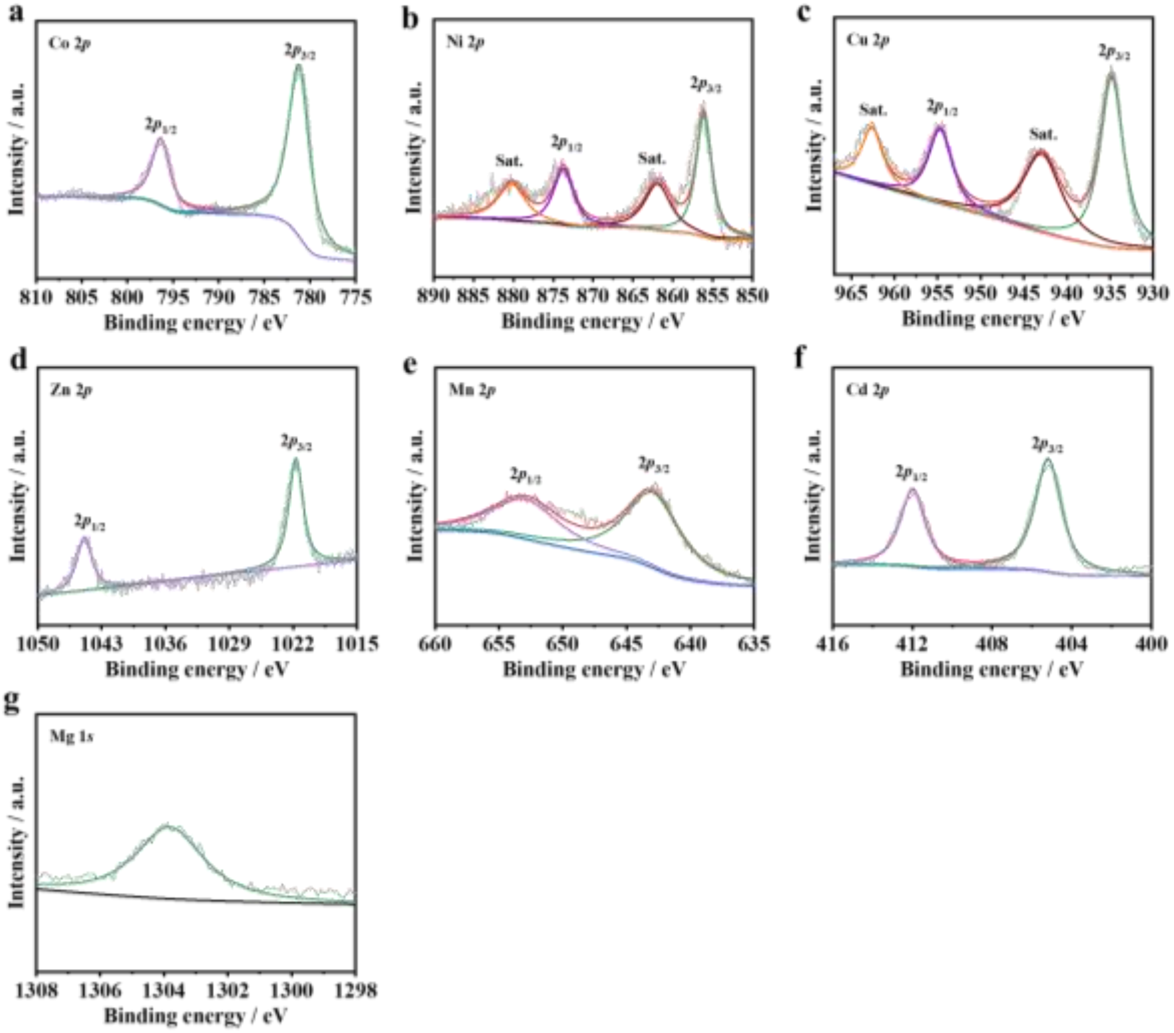

Figure S30. High-resolution (a) Co 2p, (b) Ni 2p, Cu 2p, Zn 2p, Mn 2p, Cd 2p and Mg 1s XPS spectra of CC@LHA(7) after the electrocatalytic test.

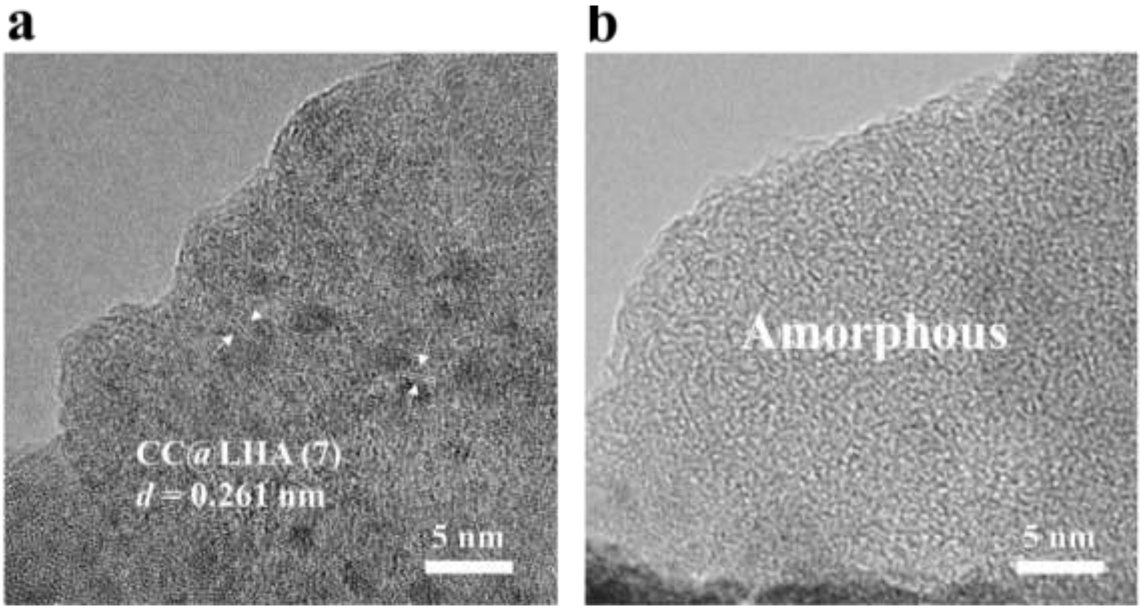

Figure S31. HRTEM images of (a) the fresh CC@LHA(7) and (b) the used CC@LHA(7) after the electrocatalytic test.

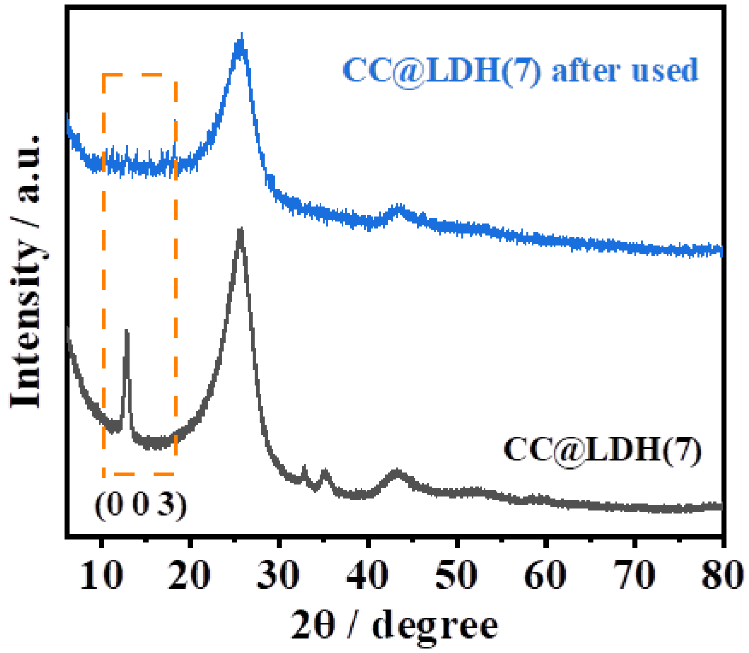

Figure S32. XRD patterns of (a) the fresh CC@LHA(7) and (b) the used CC@LHA(7) after the electrocatalytic test.

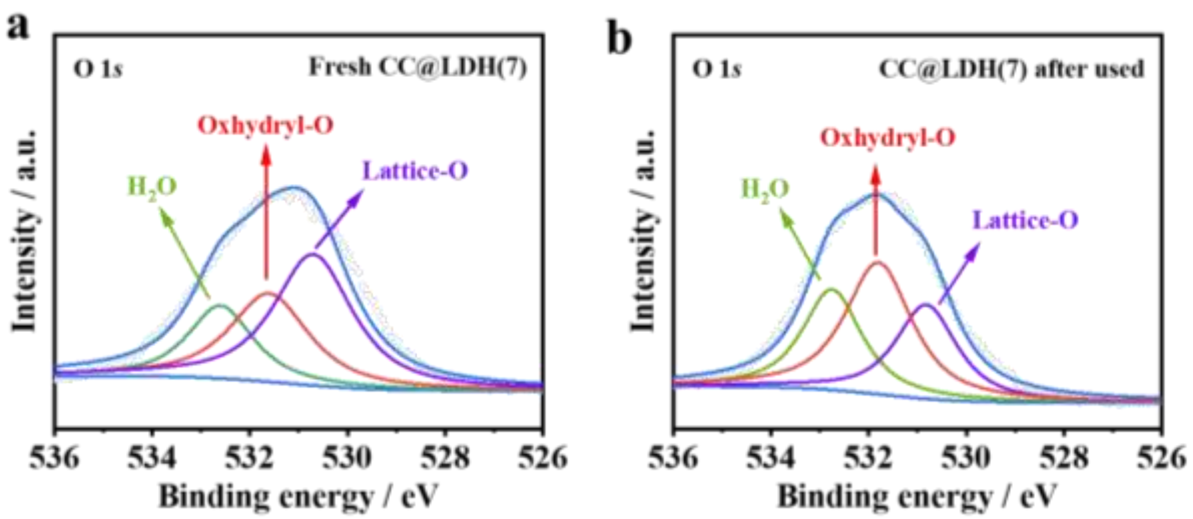

Figure S33. High-resolution O 1s XPS spectra of (a) the fresh CC@LHA(7) and (b) the used CC@LHA(7) after the electrocatalytic test.

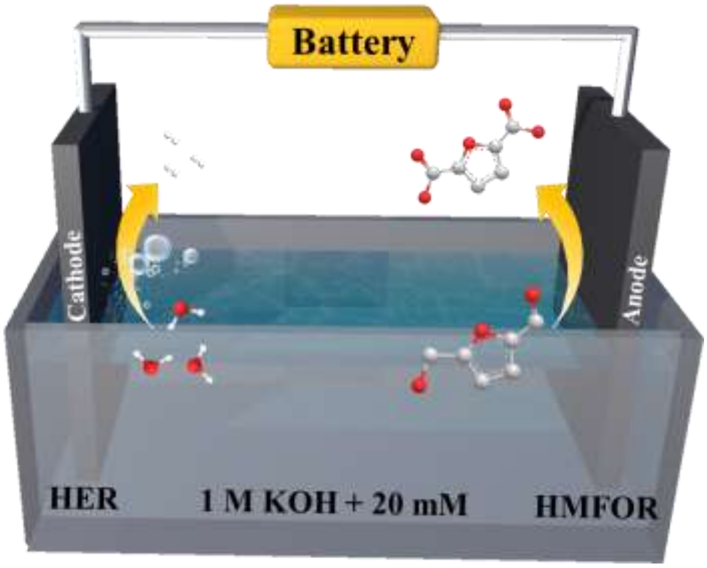

Figure S34. The schematic image of the HMFOR system coupled with HER.

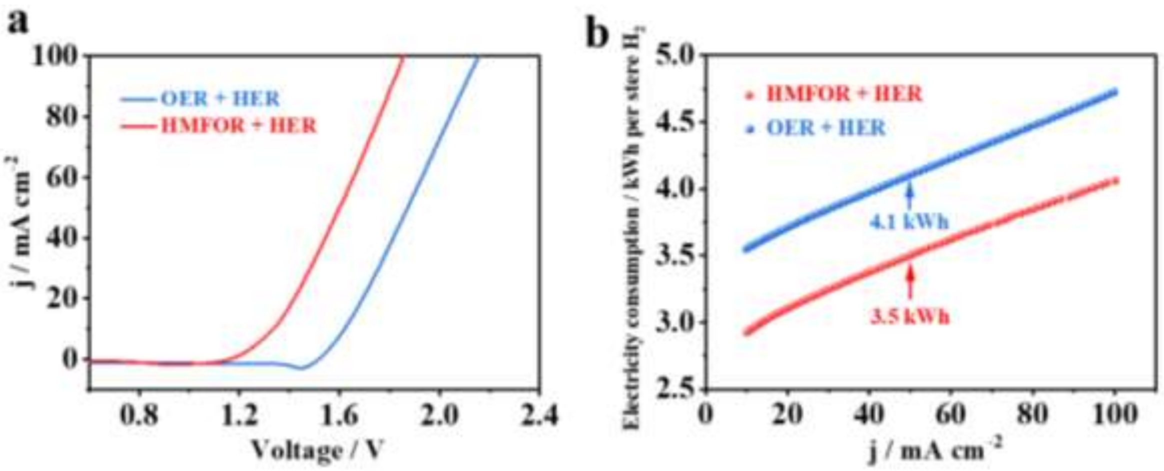

Figure S35 (a) LSV curves and (b) corresponding electricity consumptions of the two-electrode tests for HMFOR coupled HER cell and overall water splitting cell by using CC@LHA(7) and Pt foil as an anode and a cathode, respectively.

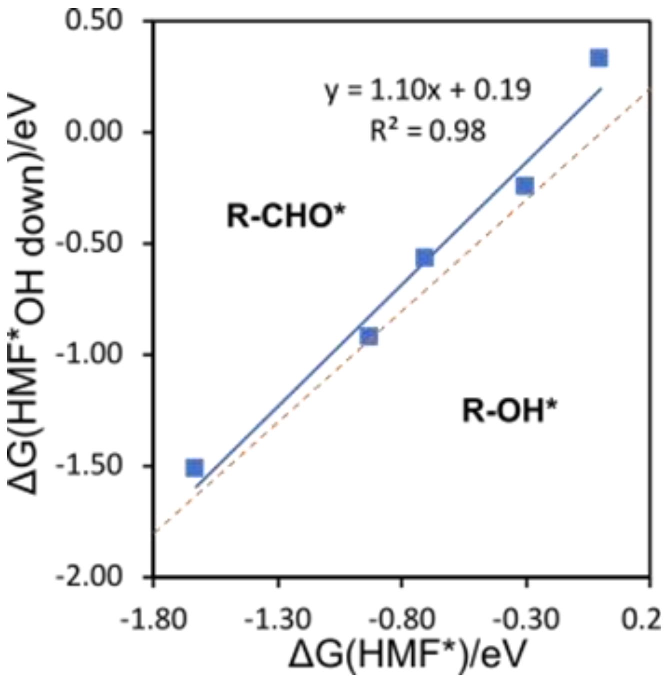

Figure S36. The scaling relation between the free energy of HMF* adsorbed via the OH* group against that via the CHO group.

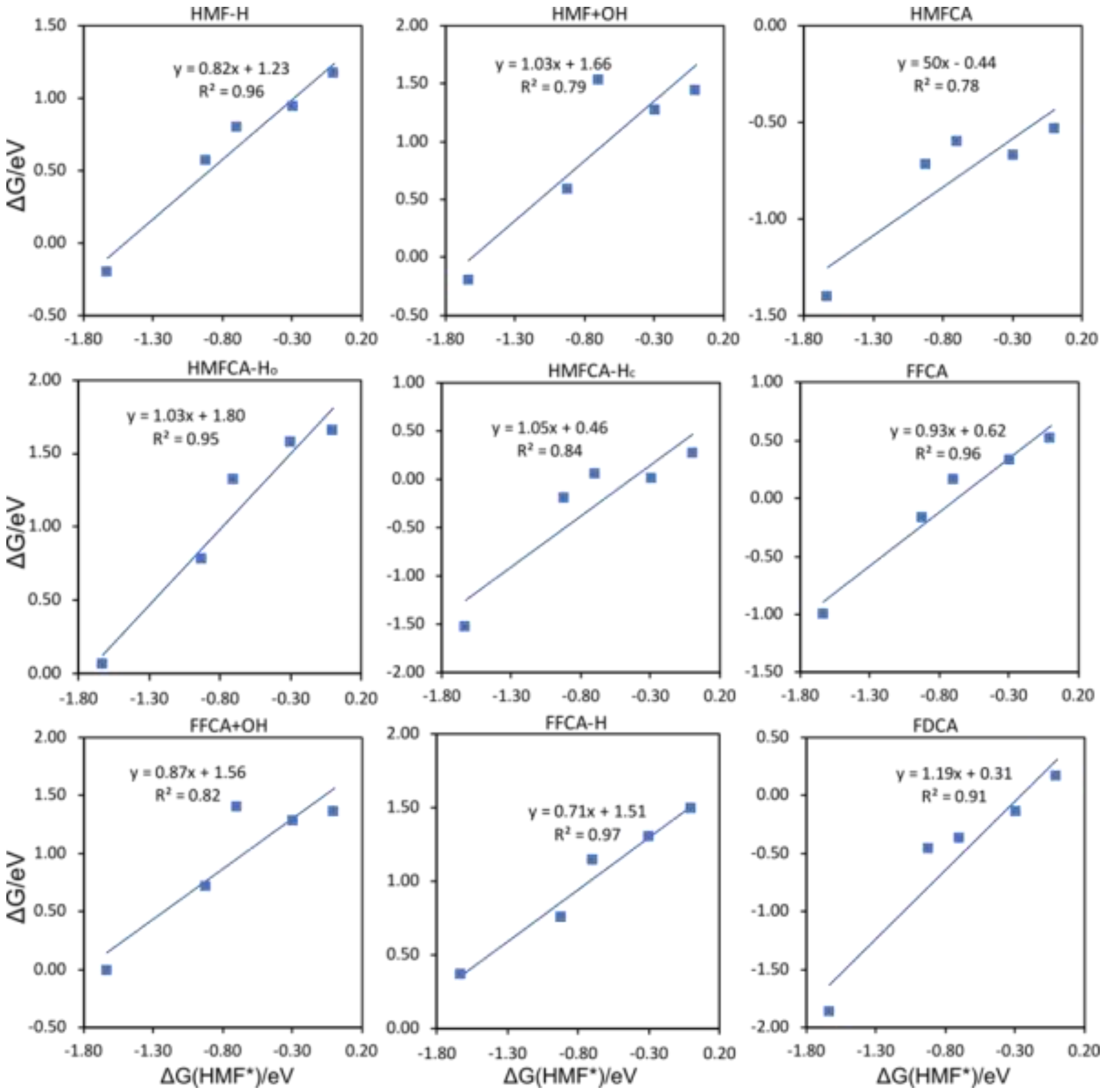

Figure S37. The scaling relationship between the energies of the HMFOR intermediates and the free energy of HMF*.

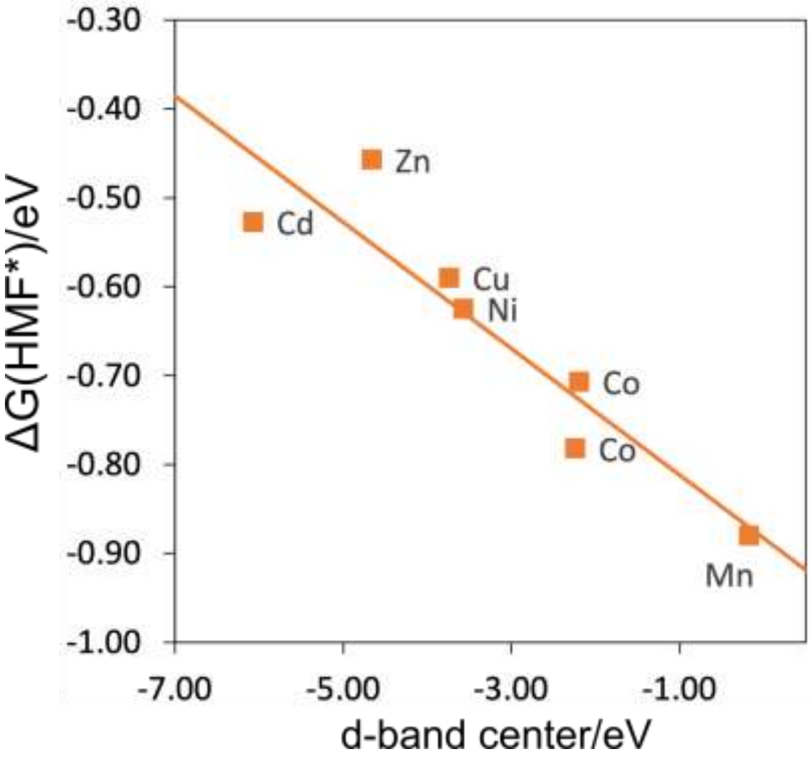

Figure S38. The scaling relationship between the free energy of the HMF* against the d-band centers.

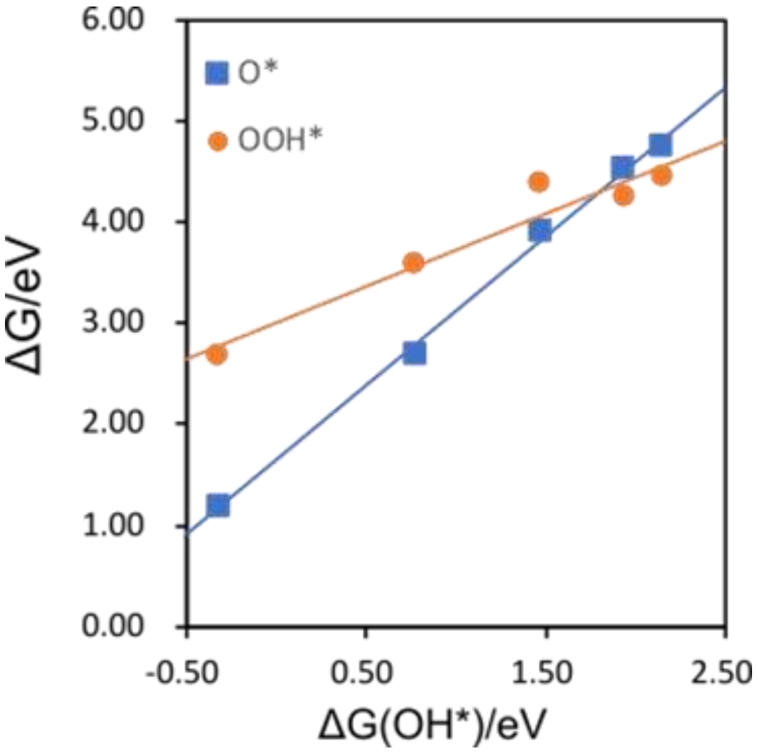

Figure S39. The scaling relation of the free energy of O* and OOH* against the free energy of OH*.

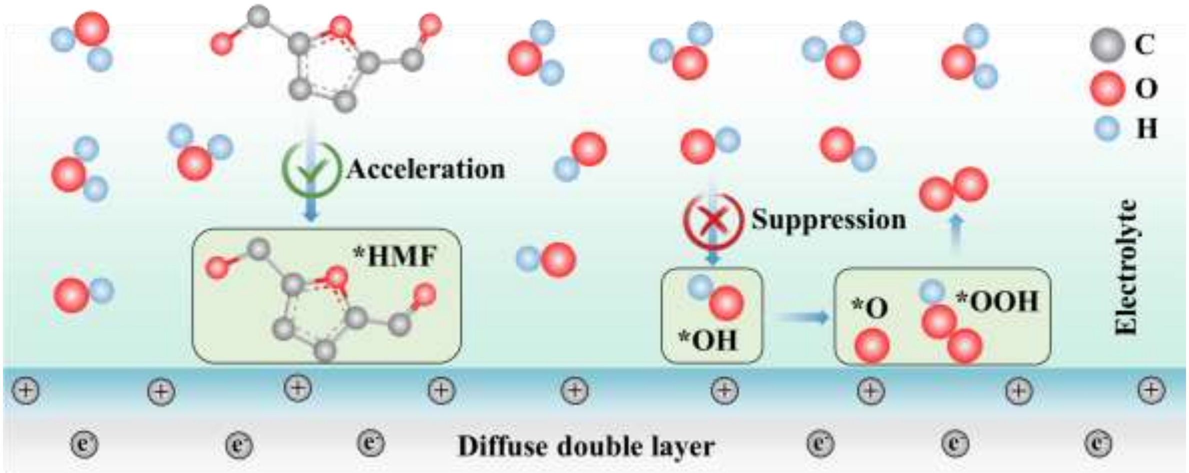

Figure S40. The schematic illustration of intermediates evolution over CC@LHA(7) during HMFOR and OER.

Table S1. The solubility product constant (K_{sp}) data of various hydroxides.

**Materials**	K_{sp}
$Mg(OH)_2$	1.8×10^{-11}
$Mn(OH)_2$	1.1×10^{-13}
$Ni(OH)_2$	2.0×10^{-15}
$Co(OH)_2$	1.6×10^{-15}
$Zn(OH)_2$	7.1×10^{-18}
$Cd(OH)_2$	2.2×10^{-14}
$Cu(OH)_2$	5.0×10^{-20}

Table S2. The crystal data of various hydroxides from their XRD standard spectra.

Materials	PDF data	Space group	a/Å	b/Å	c/Å	α/°	β/°	γ/°
$Mg(OH)_2$	PDF#78-0316	P-3m1	3.142	3.142	4.766	90	90	120
$Mn(OH)_2$	PDF#73-1133	P-3m1	3.322	3.322	4.734	90	90	120
$Ni(OH)_2$	PDF#73-1520	P-3m1	3.114	3.114	4.617	90	90	120
$Co(OH)_2$	PDF#74-1057	P-3m1	3.173	3.173	4.640	90	90	120
$Zn(OH)_2$	PDF#72-2032	P-3m1	3.194	3.194	4.714	90	90	120
$Cd(OH)_2$	PDF#13-0226	P-3m1	3.496	3.496	4.702	90	90	120
$Cu(OH)_2$	PDF#72-0140	C-mcm	2.949	1.059	5.256	90	90	90

Table S3. The ionic radius of different metallic elements with high-spin 6 coordination.

**Elements**	**Ni^{2+}**	**Mg^{2+}**	**Cu^{2+}**	**Zn^{2+}**	**Co^{2+}**	**Mn^{2+}**	**Cd^{2+}**
Ionic radius/**Å**	0.69	0.72	0.73	0.74	0.75	0.83	0.95

*All the ionic radius value are come from Shannon's work at the website of http://abulafia.mt.ic.ac.uk/shannon/ptable.php#opennewwindow.

Table S4. The net Bader charge of the ions in the LHAs

Elements	Co	Ni	Cu	Zn	Mn	Cd
LHA(7)	1.30	1.249	1.121	1.301	1.463	1.265
LHA(6)	1.29	1.247	1.131	1.331	1.346	1.269
LHA(2)	1.30	1.244	\	\	\	\

[a]A positive value means the ion is positively charged.

Table S5. The contents of different elements in CC@LHA(7) before and after the electrocatalytic test.[a]

Elements/%	Cd	Mn	Co	Ni	Cu	Zn	Mg
Fresh sample	4.81	5.11	12.83	14.49	30.19	31.34	1.22
Used sample	6.23	6.22	16.55	19.60	34.89	14.74	1.77

[a]The element contents was measured through AAS.

Table S6. Comparison of the HMFOR performances of CC@HE-LDH (7) with various previously reported electrocatalysts.

Electrocatalysts	Potential / V (current density)	FDCA selectivity	Stability	References
CC@LHA(7)	1.38 (50)	99%	10 cycles	This work
δ-MnO_2	1.4 (10)	98%	5 cycles	Ref. 19
Co-P/Cu foam	1.38 (20)	90%	1.5 h	Ref. 20
Ir/Co_3O_4	1.48 (20)	98%	6 cycles	Ref. 21
Pt/$Ni(OH)_2$	1.50 (37)	98.7 %	/	Ref. 22
N-$NiMoO_4$	1.39 (10)	91%	6 cycles	Ref. 23
NiBx	1.42 (40)	99 %	4 h	Ref. 24
MoO_2-FeP@C	1.36 (10)	98.6%	10 cycles	Ref. 25
Ni_3N-NiMoN	1.39 (50)	99%	2.5 h	Ref. 26
E-CoAl-LDH-NSA	1.59 (100)	99%	7 cycles	Ref. 27
Co_3O_4 NW/NF	1.76 (100)	97%	30 h	Ref. 28

References

(1) Kresse, G.; Furthmuller, J. Efficient Iterative Schemes for Ab Initio Total-Energy Calculations Using a Plane-Wave Basis Set. *Phys Rev B* **1996**, *54*, 11169-11186.

(2) Kresse, G.; Furthmuller, J. Efficiency of Ab-Initio Total Energy Calculations for Metals and Semiconductors Using a Plane-Wave Basis Set. *Comput Mater Sci* **1996**, *6*, 15-50.

(3) Kresse, G.; Hafner, J. Norm-Conserving and Ultrasoft Pseudopotentials for First-Row and Transition Elements. *J. Phys. Condens. Matter* **1994**, *6*, 8245.

(4) Perdew, J. P.; Burke, K.; Ernzerhof, M. Generalized Gradient Approximation Made Simple. *Phys. Rev. Lett.* **1996**, *77*, 3865-3868.

(5) Blöchl, P. E. Projector Augmented-Wave Method. *Phys. Rev. B* **1994**, *50*, 17953–17979.

(6) Dudarev, S. L.; Botton, G. A.; Savrasov, S. Y.; Humphreys, C. J.; Sutton, A. P. Electron-Energy-Loss Spectra and the Structural Stability of Nickel Oxide: An LSDA+U Study. *Phys Rev B* **1998**, *57*, 1505-1509.

(7) Chen, J.; Selloni, A. First Principles Study of Cobalt (Hydr)Oxides under Electrochemical Conditions. *J. Phys. Chem. C* **2013**, *117*, 20002-20006.

(8) Tkalych, A. J.; Yu, K.; Carter, E. A. Structural and Electronic Features of β-$Ni(OH)_2$ and β-NiOOH from First Principles. *J. Phys. Chem. C* **2015**, *119*, 24315-24322.

(9) Wang, L.; Maxisch, T.; Ceder, G. Oxidation Energies of Transition Metal Oxides within the GGA + U Framework. *Phys. Rev. B* **2006**, *73*, 195107.

(10) Liu, X.; Schlexer, P.; Xiao, J.; Ji, Y.; Wang, L.; Sandberg, R. B.; Tang, M.; Brown, K. S.; Peng, H.; Ringe, S.; Hahn, C.; Jaramillo, T. F.; Nørskov, J. K.; Chan, K. pH Effects on the Electrochemical Reduction of CO_2 towards C2 Products on Stepped Copper. *Nat. Commun.* **2019**, *10*, 32.

(11) Bajdich, M.; García-Mota, M.; Vojvodic, A.; Nørskov, J. K.; Bell, A. T. Theoretical Investigation of the Activity of Cobalt Oxides for the Electrochemical Oxidation of Water. *J. Am. Chem. Soc.* **2013**, *135*, 13521-13530.

(12) Shin, H.; Xiao, H.; Goddard, W. A. In Silico Discovery of New Dopants for Fe-Doped Ni Oxyhydroxide (Ni1-xFexOOH) Catalysts for Oxygen Evolution Reaction. *J. Am. Chem. Soc.* **2018**, *140*, 6745-6748.

(13) Monkhorst, H. J.; Pack, J. D. Special Points for Brillouin-Zone Integrations. *Phys. Rev. B* **1976**, *13*, 5188-5192.

(14) Mathew, K.; Sundararaman, R.; Letchworth-Weaver, K.; Arias, T. A.; Hennig, R. G. Implicit Solvation Model for Density-Functional Study of Nanocrystal Surfaces and Reaction Pathways. *J. Chem. Phys.* **2014**, *140*, 084106.

(15) Grimme, S.; Antony, J.; Ehrlich, S.; Krieg, H. A Consistent and Accurate Ab Initio Parametrization of Density Functional Dispersion Correction (DFT-D) for the 94 Elements H-Pu. *J. Chem. Phys.* **2010**, *132*, 154104.

(16) Grimme, S.; Ehrlich, S.; Goerigk, L. Effect of the Damping Function in Dispersion Corrected Density Functional Theory. *J. Comput. Chem.* **2011**, *32*, 1456-1465.

(17) Nørskov, J. K.; Rossmeisl, J.; Logadottir, A.; Lindqvist, L.; Kitchin, J. R.; Bligaard, T.; Jónsson, H. Origin of the Overpotential for Oxygen Reduction at a Fuel-Cell Cathode. *J. Phys. Chem. B* **2004**, *108*, 17886-17892.

(18) Peterson, A. A.; Abild-Pedersen, F.; Studt, F.; Rossmeisl, J.; Nørskov, J. K. How Copper Catalyzes the Electroreduction of Carbon Dioxide into Hydrocarbon Fuels. *Energy Environ. Sci.* **2010**, *3*, 1311-1315.

(19) Wang, C.; Bongard, H.-J.; Weidenthaler, C.; Wu, Y.; Schüth, F. Design and Application of a High-Surface-Area Mesoporous δ-MnO_2 Electrocatalyst for Biomass Oxidative Valorization. *Chem. Mater.* **2022**, *34*, 3123-3132.

(20) Jiang, N.; You, B.; Boonstra, R.; Rodriguez, I. M. T.; Sun, Y. Integrating Electrocatalytic 5-Hydroxymethylfurfural Oxidation and Hydrogen Production via Co-P-Derived Electrocatalysts. *ACS Energy Lett.* **2016**, *1*, 386-390.

(21) Lu, Y.; Liu, T.; Dong, C.L.; Huang, Y.C.; Li, Y.; Chen, J.; Zou, Y.; Wang, S. Tuning the Selective Adsorption Site of Biomass on Co_3O_4 by Ir Single Atoms for Electrosynthesis. *Adv. Mater.* **2021**, *33*, 2007056.

(22) Zhou, B.; Li, Y.; Zou, Y.; Chen, W.; Zhou, W.; Song, M.; Wu, Y.; Lu, Y.; Liu, J.; Wang, Y.; Wang, S. Platinum Modulates Redox Properties and 5-Hydroxymethylfurfural Adsorption Kinetics of $Ni(OH)_2$ for Biomass Upgrading. *Angew. Chem. Int. Ed.* **2021**, *60*, 22908-22914.

(23) Wang, W.; Wang, M. Nitrogen Modulated $NiMoO_4$ with Enhanced Activity for the Electrochemical Oxidation of 5-Hydroxymethylfurfural to 2,5-Furandicarboxylic Acid. *Catal. Sci. Technol.* **2021**, *11*, 7326-7330.

(24) Zhang, P.; Sheng, X.; Chen, X.; Fang, Z.; Jiang, J.; Wang, M.; Li, F.; Fan, L.; Ren, Y.; Zhang, B.; Timmer, B. J. J.; Ahlquist, M. S. G.; Sun, L. Paired Electrocatalytic Oxygenation and Hydrogenation of Organic Substrates with Water as the Oxygen and Hydrogen Source. *Angew. Chem. Int. Ed.* **2019**, *58*, 9155-9159.

(25) Yang, G.; Jiao, Y.; Yan, H.; Xie, Y.; Wu, A.; Dong, X.; Guo, D.; Tian, C.; Fu, H. Interfacial Engineering of MoO2-FeP Heterojunction for Highly Efficient Hydrogen Evolution Coupled with Biomass Electrooxidation. *Adv. Mater.* **2020**, *32*, 2000455.

(26) Zeng, J.; Chen, W.; Zhang, G.; Yu, L.; Zhong, L.; Liu, Y.; Zhao, S.; Qiu, Y. Heterostructured Ni3N–NiMoN Nanowires as Bifunctional Electrocatalysts for Hydrogen Evolution and 5-Hydroxymethylfurfural Oxidation. *ACS Appl. Nano Mater.* **2022**, *5*, 7321-7330.

(27) Song, Y.; Li, Z.; Fan, K.; Ren, Z.; Xie, W.; Yang, Y.; Shao, M.; Wei, M. Ultrathin Layered Double Hydroxides Nanosheets Array towards Efficient Electrooxidation of 5-Hydroxymethylfurfural Coupled with Hydrogen Generation. *Appl. Catal. B* **2021**, *299*, 120669.

(28) Zhou, Z.; Chen, C.; Gao, M.; Xia, B.; Zhang, J. In Situ Anchoring of a Co_3O_4 Nanowire on Nickel Foam: an Outstanding Bifunctional Catalyst for Energy-Saving Simultaneous Reaction. *Green Chem.* **2019**, *21*, 6699-6706.
